# Pathological insights into cerebral amyloid angiopathy underlying intracerebral haemorrhage: population-based autopsy study

**DOI:** 10.1007/s00401-026-02980-0

**Published:** 2026-01-24

**Authors:** Ya Su, Mark A. Rodrigues, Neshika Samarasekera, James J. M. Loan, Alice Hosking, Tom J. Moullaali, Catherine A. Humphreys, Karina McDade, Tracey Millar, Joanna M. Wardlaw, Xin Cheng, Susanne J. van Veluw, Rustam Al-Shahi Salman, Colin Smith

**Affiliations:** 1 Centre for Clinical Brain Sciences, Institute for Neuroscience and Cardiovascular Research, The University of Edinburgh, Chancellor’s Building, 49 Little France Crescent, Edinburgh, EH16 4SB UK; 2 Department of Neurology, National Centre for Neurological Disorders, National Clinical Research Centre for Aging and Medicine, Huashan Hospital, Fudan University, Shanghai, China; 3 Department of Neuroradiology, NHS Lothian, Edinburgh, UK; 4 Edinburgh Imaging, The University of Edinburgh, Edinburgh, UK; 5 BHF-UKDRI Centre for Vascular Dementia Research, The University of Edinburgh, Edinburgh, UK

**Keywords:** Cerebral Amyloid Angiopathy, Intracerebral Haemorrhage, Post-mortem Autopsy, Pathology

## Abstract

Lobar intracerebral haemorrhage (ICH) is associated with cerebral amyloid angiopathy (CAA) pathology. Uncertainty remains about the mechanisms leading from CAA to ICH. We investigated the distribution and characteristics of CAA, and its clinical and neuropathological associations. Participants underwent research autopsy in the Lothian IntraCerebral Haemorrhage, Pathology, Imaging and Neurological Outcome (LINCHPIN) study. Neuropathologists rated tissue for CAA using standardised consensus criteria, as well as non-amyloid small vessel disease, Thal phase, and Braak stage. We compared the presence, distribution, and severity of CAA among different brain regions, and in the lobe or hemisphere affected by lobar ICH to corresponding contralateral regions. We evaluated the diagnostic accuracy of Vonsattel CAA grade on a post-mortem cortical specimen (simulating surgical biopsy) versus the reference standard of moderate-to-severe parenchymal CAA at autopsy. Among 162 participants, parenchymal CAA, meningeal CAA, and CAA-associated vasculopathy were diffusely distributed among all cerebral lobes irrespective of the ICH location, but capillary CAA showed an occipital predominance. In lobar ICH, all CAA measures did not differ between the ICH lobe or hemisphere and the contralateral unaffected region. CAA measures did not increase with age, but they were higher in carriers of *APOE* ε2 or ε4 alleles and in individuals with higher Thal phase or Braak stage. Using a rule-out category of Vonsattel grade ≥ 1 to diagnose CAA on a simulated cortical biopsy achieved 100% sensitivity (95%CI 93.4–100), and a rule-in category of Vonsattel grade ≥ 2 had 79.5% specificity (95%CI 63.5–90.7) versus the reference standard. The distribution and severity of parenchymal CAA, meningeal CAA, and CAA-associated vasculopathy are diffuse regardless of ICH location, indicating the need to better understand the factors underlying bleeding in CAA-affected vessels.

**Supplementary Information:**

The online version contains supplementary material available at 10.1007/s00401-026-02980-0.

## Introduction

Arteriolosclerosis and cerebral amyloid angiopathy (CAA) are the two most common forms of sporadic small vessel disease (SVD), together accounting for approximately 85% of all spontaneous intracerebral haemorrhages (ICH) [1, 2]. Characterising the severity and spatial distribution of presumed SVD pathology in ICH contributes to understanding how SVD leads to SVD-related ICH. Histopathological examination on post-mortem autopsy remains the reference standard for identifying the underlying causes of ICH and for validating in vivo neuroimaging diagnostic criteria of SVD, as it enables comprehensive multi-regional tissue analyses [3].

CAA, a major risk factor for lobar ICH [4], is neuropathologically characterised by *β*-amyloid (A*β*) deposition in small cortical and leptomeningeal vessels and may have an occipital predominance [5–7]. Through a systematic review, 29 case series have provided a clear histopathological definition for CAA and described the severity or distribution of CAA in ICH (details in Supplementary Material 1). However, most previous studies were retrospective, hospital-based, non-consecutive convenience samples, potentially limiting generalisability to the general ICH population. Moreover, many studies did not apply systematic pathological assessment methods for CAA, and showed inconsistent findings regarding the distribution of CAA. To date, more than thirty different staging systems have been used to evaluate CAA pathology; some of these focus solely on the percentage or number of affected vessels per cortical area, limiting their ability to reflect the global CAA pathology [8]. Vonsattel and Love scales represent the most widely applied criteria for grading both CAA severity and vasculopathy on pathology [9, 10]. Vonsattel grading relates to intrinsic vessel wall pathology, from histologically normal vessels with a rim of A*β* around smooth muscle fibres to extensive vessel wall scarring with intermingled A*β*, whereas Love further distinguishes distinct vessel types affected by CAA and assesses the burden of pathology across multiple regions of the whole brain, providing more detailed information on the severity and distribution of CAA [10].

The mechanisms by which CAA leads to ICH remain incompletely understood. Previous pathological studies have linked CAA-ICH to more severe vascular A*β* deposition [11], whereas others have emphasised the role of secondary vasculopathic changes, such as microaneurysms and fibrinoid necrosis [9, 12]. In addition, Apolipoprotein E (*APOE*) ε2 and ε4 genotypes may contribute to ICH risk [13, 14]. A detailed characterisation of CAA severity and associated vasculopathy, along with their comparisons between ICH-affected versus unaffected brain regions, could provide valuable insights.

Understanding whether CAA pathology is diffusely distributed or more severe at the ICH site is, therefore, directly relevant to the clinical question of the reliability of a small cortical biopsy sample for assessing the presence and severity of CAA in ICH. Hematoma evacuation or biopsy offers the opportunity to obtain cortical specimens to identify the etiology of ICH. However, diagnostic accuracy may be limited by the patchy distribution of CAA and the limited tissue volume obtained [15], as well as by incidental age-related CAA pathology in the elderly [9]. So far, only one study, including 28 post-mortem specimens, which were used as a simulated biopsy from two participants with CAA-ICH and without non-CAA ICH controls evaluated the diagnostic accuracy of biopsy, reporting 100% sensitivity and 95–77% specificity with advancing age for Vonsattel grade ≥ 1 [9].

Therefore, we aimed to characterise in detail CAA presence, distribution, and severity in individuals with lobar ICH compared to non-lobar ICH from a population-based ICH cohort, to help advance our understanding of CAA and the relationship with lobar ICH. Additionally, we used a post-mortem cortical specimen containing the ICH epicentre as a simulated cortical biopsy, and assessed the diagnostic accuracy of simulated cortical biopsies against the whole brain as a reference standard to inform whether surgical biopsy can reliably diagnose CAA-related ICH.

## Materials and methods

### Study design and participants

We conducted a cross-sectional study from the prospective population-based inception cohort study of adults (aged ≥ 16 years) with spontaneous ICH in the Lothian health board region of Scotland (the Lothian IntraCerebral Haemorrhage, Pathology, Imaging and Neurological Outcome [LINCHPIN] study), who were enrolled between 1 June 2010 and 31 March 2023 [16]. Autopsy samples were collected up to 1 March 2025. Consecutive participants were prospectively identified using multiple overlapping sources of case ascertainment and confirmed by a CT brain scan [2]. Exclusion criteria included exclusively extra-axial intracranial haemorrhage, ICH secondary to trauma, macrovascular/structural causes, or haemorrhagic transformation of ischaemic stroke. Demographics, medical history, and medication data were collected via patient/family/carer interviews and medical record reviews. The cognitive status of unimpaired cognition, mild cognitive impairment, and dementia was determined by DSM-V criteria as previously described [17].

We aimed to recruit consecutive adults for research autopsy (limited to the brain) in the event of death, to study the nature of SVD in adults with ICH [16]. If the patient lacked mental capacity as defined by the Adults with Incapacity (Scotland) Act 2000, we sought consent for brain tissue donation from their nearest relative or legal representative in accordance with the statutory requirements of the Human Tissue (Scotland) Act 2006. This study was approved by the Scotland A Research Ethics Committee (10/MRE00/23) and carried out in accordance with the Declaration of Helsinki. The written informed consents were provided by all participants or their next-of-kin.

### Neuroimaging evaluations

One neuroradiologist (MAR) evaluated diagnostic brain CT images, blinded to the clinical and neuropathological findings, with a standardised proforma derived from large-scale stroke studies as previously described [18]. MAR evaluated the ICH location using the Cerebral Haemorrhage Anatomical RaTing Scale (CHARTS) [19] and calculated the ICH volume using the ABC/2 method [20]. The ICH location was classified as either ‘lobar’ or ‘non-lobar’ as previously described [16]. ‘Non-lobar’ ICH was defined if there was a single infratentorial ICH (located in the brainstem or cerebellum), a single supratentorial deep ICH (located in the basal ganglia, internal or external capsule, or thalamus without extension to a lobar area), or multiple ICHs in solely non-lobar locations. All other ICHs were ‘lobar’.

MAR assessed extra-axial haemorrhage (in the subarachnoid, subdural, or intra-ventricular spaces), finger-like projections, and the severity of anterior and posterior white matter lucencies, lacunes, and central and cortical atrophy [18, 21].

### Research brain autopsy and neuropathological assessment

Two neuropathologists (CS and CAH) systematically evaluated formalin-fixed post-mortem brain tissue following a standardised protocol as previously described [18, 22]. The maximum interval from death to autopsy was 5 days. CAA, A*β* plaques, and tau were sought using immunohistochemistry with a monoclonal mouse antibody to human Aβ (clone 6F/3D, Dako, Denmark) at a concentration of 1:100 and a monoclonal mouse antibody to human tau (clone AT8, Thermo Fisher Scientific, USA) at a concentration of 1:2500 respectively. Investigators were blinded to CT imaging, clinical data, and ICH location when possible (unless the ICH was included in one of the prespecified sampled regions) in microscopic histopathological assessment.

**Fig. 1:**
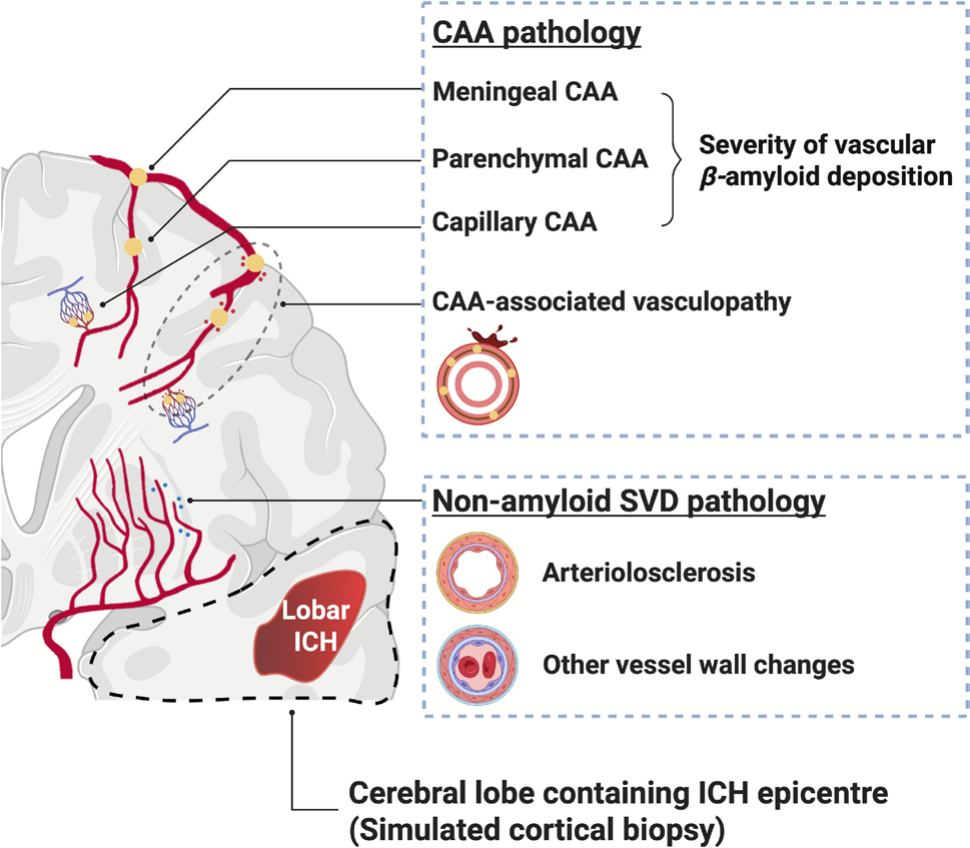
Schematic diagram of the histopathological grading system for CAA and non-amyloid SVD, and the simulated cortical biopsy on post-mortem specimens. CAA pathology is graded using the Love scale. It quantifies the severity of vascular *β*-amyloid deposition, including parenchymal arterial CAA (0–3), meningeal arterial CAA (0–3), and capillary CAA (0–1), as well as the severity of secondary CAA-associated vasculopathy (0–2), which is characterised by thrombosis, micro-haemorrhage, concentric splitting, and fibrinoid necrosis of *β*-amyloid-laden vessels. Non-amyloid SVD pathology of arteriolosclerosis, lipohyalinosis, and fibrinoid necrosis is rated with a 4-point scale. The simulated cortical biopsy is taken from the post-mortem cortical specimen containing the ICH epicentre. CAA = cerebral amyloid angiopathy; SVD = small vessel disease; ICH = intracerebral haemorrhage

Detailed procedures and scales for neuropathological assessments are shown in Fig. 1 and Supplementary Material 2. For SVD, both CAA and non-amyloid SVD pathology were assessed. CS rated CAA features in all cerebral (frontal, temporal, parietal, and occipital) and cerebellar lobes using a validated consensus scale developed by Love et al. [10].It quantified the severity of vascular A*β* deposition, including parenchymal arterial CAA (0–3), meningeal arterial CAA (0–3), and capillary CAA (0–1), as well as the severity of secondary CAA-associated vasculopathy (0–2), which was characterised by thrombosis, micro-haemorrhage, concentric splitting, and fibrinoid necrosis of A*β*-laden vessels. CS and CAH rated the global severity of non-amyloid SVD features of arteriolosclerosis, lipohyalinosis, and fibrinoid necrosis using haematoxylin and eosin-stained sections with a modified version of a published 4-point scale (0 = none, 1 = mild, 2 = moderate, 3 = severe) [23]. Non-amyloid SVD features were only assessed in the left hemisphere, since it was usually considered symmetrical [3, 22]. For non-SVD neurodegenerative pathology, CS rated burdens of A*β* plaques and neurofibrillary tangles using validated scales of Thal phase (0–5 depending on A*β* plaque distribution, from none, to neocortex, limbic system, subcortical nuclei, brainstem and finally cerebellar involvement; CAA was not included in this score) [24] and Braak stage (0-VI determined by neurofibrillary tangle distribution, from none to transentorhinal, limbic regions and finally extensive neocortical involvement) respectively. [25, 26] Inter-rater reliabilities for pathological assessment were substantial to almost perfect (Cohen’s kappa: CAA score, 0.976; non-amyloid SVD, 0.663; Thal phase, 0.654; Braak stage, 0.725). Discrepancies were resolved by consensus.

For global CAA severity, we made the CAA ratings similar to the non-amyloid SVD ratings by summing the parenchymal CAA scores for both cerebral hemispheres and grading for a CAA burden category (0 = none, 1–8 = mild, 9–16 = moderate, and 17–24 = severe). Definite CAA was defined as moderate-to-severe parenchymal CAA plus the presence of CAA-associated vasculopathy, a pathology-based definition conceptually aligned with the ‘definite CAA’ category in the Boston criteria v2.0 [27]. The Vonsattel scale for grading CAA was defined by the most advanced degree of parenchymal or meningeal CAA present within the specimen (0, 1, and ≥ 2) [9]. The histopathological diagnoses for ICH were categorised as follows: none (neither moderate-to-severe CAA nor non-amyloid SVD), pure CAA (moderate-to-severe CAA alone), pure non-amyloid SVD (moderate-to-severe non-amyloid SVD alone), and mixed CAA and non-amyloid SVD (moderate-to-severe CAA and non-amyloid SVD together) [18].

### Simulated cortical biopsy for lobar ICH

We used a post-mortem cortical specimen containing the ICH epicentre as a simulated cortical biopsy (Fig. 1). For participants with multiple simultaneous ICH lesions, the largest supratentorial ICH lesion would be selected, as it was likely to be the preferred site for hematoma evacuation. We did not perform additional tissue sampling. Instead, we used the prespecified cortical regions routinely sampled for neuropathological assessment in the ICH-affected lobe, which allowed us to assess the anatomical area most relevant to the ICH lesion and to comprehensively evaluate all CAA measures.

### *APOE* genotype

We obtained DNA from peripheral blood samples or cerebellar tissue stored in the LINCHPIN brain bank with the standard methods described [18]. We classified the *APOE* genotype as *APOE* ε2 or ε4 possession if participants had at least one ε2 or ε4 allele. Participants with *APOE* ε2/ε4 were therefore included in both groups.

### Statistical analysis

The sample size was determined by the maximum number of available participants meeting the requirements for autopsy data. No *APOE* genotype or pathological data were missing in this study. For descriptive statistics, we used one-way analysis of variance or Mann–Whitney *U* test, and χ2 test or Fisher’s exact test to compare groups.

Binary or ordinal logistic regression models adjusted for age, sex, and time interval from index ICH to autopsy were used to estimate the intergroup differences in all pathological scores between participants with lobar and non-lobar ICH, with the false discovery rate (FDR) method applied to correct for multiple comparisons.

For the distribution of CAA pathology, pathological scores across all five regions were compared by generalised estimating equations (GEE) to account for intra-individual correlations, using ordinal regression for scores with ≥ 3 categories and binomial regression for binary scores. Multiple post-hoc pairwise comparisons were adjusted for the FDR method. To assess the frequency of CAA severity in the ICH-affected versus ICH-unaffected hemisphere, and in the ICH-affected lobe (i.e. the lobe containing the ICH epicentre) versus the contralateral homologous ICH-unaffected lobe, we used the paired Mcnemar’s or Stuart-Maxwell tests for comparisons. We conducted prespecified analyses to examine trends in CAA severity and distribution across subgroups stratified by age tertiles at the time of the index ICH, by Thal phase (Thal 0–1, 2–3, 4–5), and by Braak stage (Braak 0-II, III-IV, V-VI) using Cuzick’s nonparametric trend test, and differences between *APOE* ε2 or ε4 carriers and non-carriers using the Mann–Whitney *U* test, based on prior evidence showing that CAA severity increased with advancing age [9], more severe AD pathology [8], and *APOE* ε2 or ε4 carrier status [13].

We evaluated the accuracy of diagnosing CAA presence in the simulated biopsy (Vonsattel grade of ≥ 1 or ≥ 2) against the reference standard of moderate-to-severe parenchymal CAA or definite CAA at autopsy. We also evaluated the accuracy of assessing CAA severity in the simulated biopsy, using CAA pathology severity in the whole brain as the reference standard. Diagnostic metrics included sensitivity, specificity, positive and negative predictive values, area under the receiver operating characteristic curve (AUC), and 95% confidence intervals (CI). We performed prespecified sensitivity analyses of the diagnostic accuracy for CAA presence stratified by age tertiles, given that the specificity of simulated cortical biopsies for CAA was shown to decrease with age [9]. The specificity among age groups was compared using χ2 test or Fisher’s exact test. A subgroup analysis was performed in participants with the time interval from index ICH to autopsy within one year.

Statistical Analyses were performed using R Project for Statistical Computing (version 4.4.1). A two-tailed *P* < 0.05 was considered as significant.

## Results

There were 977 participants with first-ever spontaneous ICH presumed due to SVD between 1 June 2010 and 31 March 2023. Five hundred and one consented to the LINCHPIN study, and 162 of these participants died and underwent research brain autopsy till 1 March 2025 (Supplementary Fig. 1). The median age was 81 years (interquartile range [IQR] 75–87) and 79 (49%) were men. The median time from index ICH to death was 11.5 days (IQR 4–492) and the median time from death to autopsy was 2 days (IQR 2–4). Participants who underwent research autopsy were older and more likely to have atrial fibrillation, myocardial infarction, pre-ICH cognitive impairment, and antiplatelet medication use compared with those who did not (Supplementary Table 1).

### CAA presence and its severity in lobar and non-lobar ICH

Ninety-three (57.4%) participants had lobar ICH, 58 (35.8%) had deep supratentorial ICH, and 11 (6.8%) had infratentorial ICH (including 4 brainstem ICH and 7 cerebellar ICH). There were no significant differences in the demographic and clinical characteristics of participants with lobar and non-lobar ICH. However, participants with lobar ICH had larger hematoma volume and lower brain atrophy score and lacune number compared with those with non-lobar ICH (Table 1).

**Table 1:** Baseline clinical and imaging features of participants with first-ever lobar ICH versus non-lobar ICH

	Lobar ICH (*n* = 93)	Non-lobar ICH (*n* = 69)	*P* value
Sex, male	40 (43.0)	39 (56.5)	0.089
Age at index ICH, years	82 (76, 86.5)	81 (73, 87)	0.574
Co-morbidities before ICH			
Hypertension	59 (63.4)	51 (73.9)	0.158
Diabetes mellitus	13 (14.0)	11 (15.9)	0.728
Ischaemic stroke	13 (14.0)	15 (21.7)	0.196
Atrial fibrillation	22 (23.7)	22 (31.9)	0.244
Myocardial infarction	12 (12.9)	7 (10.1)	0.590
Hyperlipidaemia	14 (15.1)	13 (18.8)	0.522
Cognitive status at ICH			0.626
Unimpaired cognition	62 (66.7)	51 (73.9)	
Mild cognitive impairment	15 (16.1)	8 (11.6)	
Dementia	16 (17.2)	10 (14.5)	
Medication at ICH			
Antiplatelet drug(s)	43 (46.2)	30 (43.5)	0.727
Anticoagulant drug(s)	13 (14.0)	13 (18.8)	0.404
Antihypertensive drug(s)	61 (65.6)	35 (50.7)	0.057
*APOE* allele status			
* APOE* ε2 carrier	25 (26.9)	10 (14.5)	0.058
* APOE* ε4 carrier	32 (34.4)	19 (27.5)	0.352
CT characteristics of index ICH^a^			
ICH volume	55.9 (18.6, 101.1)	13.3 (5.1, 31.6)	< 0.001
Intraventricular haemorrhage	43 (47.3)	44 (63.8)	0.038
Subarachnoid haemorrhage	70 (76.9)	11 (15.9)	< 0.001
Subdural haemorrhage	20 (22.0)	0	< 0.001
Finger-like projections	27 (29.7)	3 (4.3)	< 0.001
White matter lucency score	3 (1, 4)	3 (1, 4)	0.803
Brain atrophy score	2 (1, 2)	2 (2, 3)	< 0.001
Lacune number	0 (0, 0)	0 (0, 1.5)	0.001
Time from index ICH to autopsy, days	20 (7, 547)	14 (6, 387)	0.474

^a^Data missing for 2 participants with lobar ICH

Data are shown as *n* (%) for categorical variables or median (interquartile range) for non-normally distributed continuous variables

*ICH* intracerebral haemorrhage, *APOE* apolipoprotein E, *SVD* small vessel disease

**Fig. 2:**
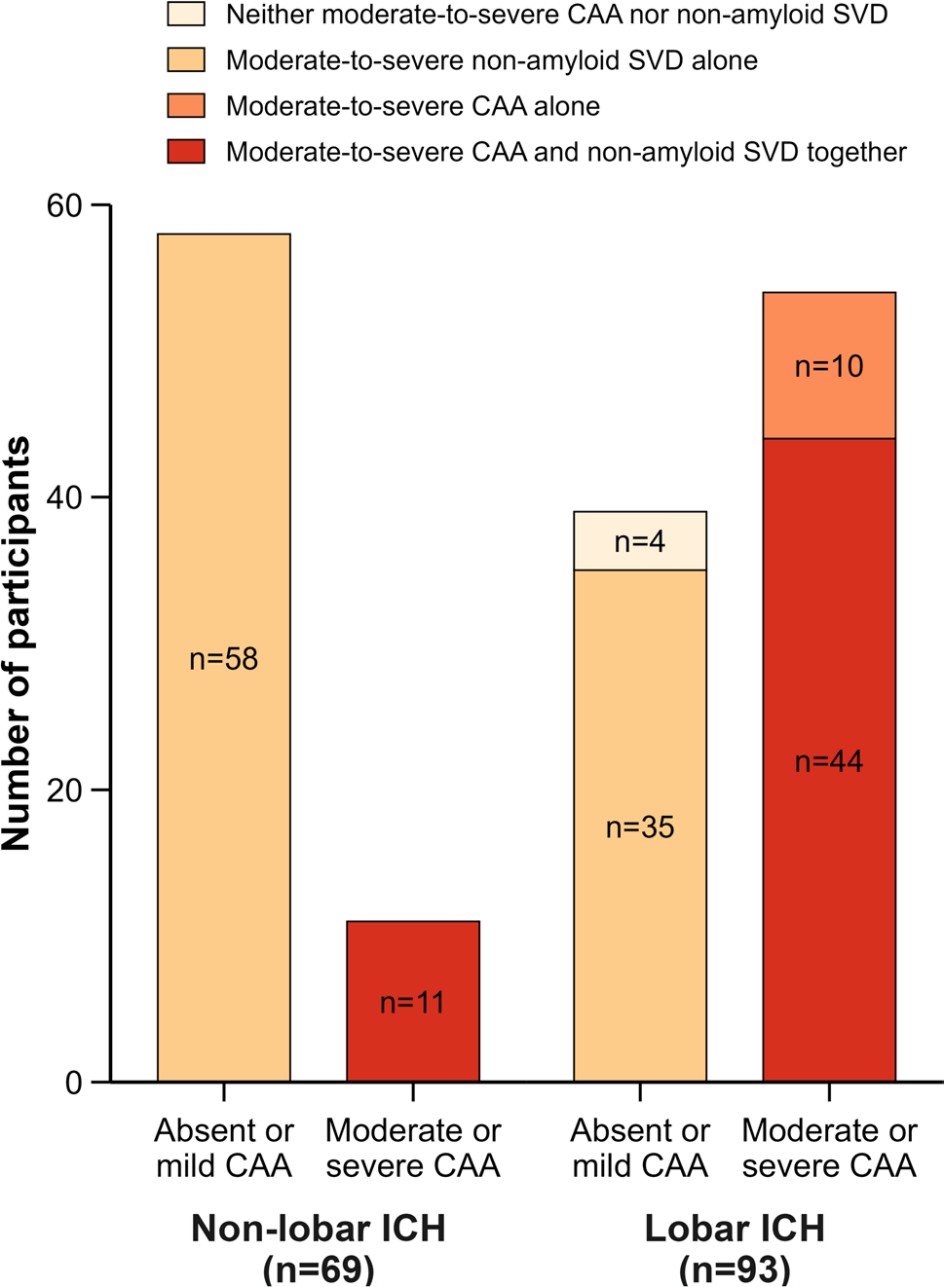
Histopathological diagnosis classification of CAA and non-amyloid SVD according to ICH location. CAA = cerebral amyloid angiopathy; SVD = small vessel disease; ICH = intracerebral haemorrhage

**Fig. 3:**
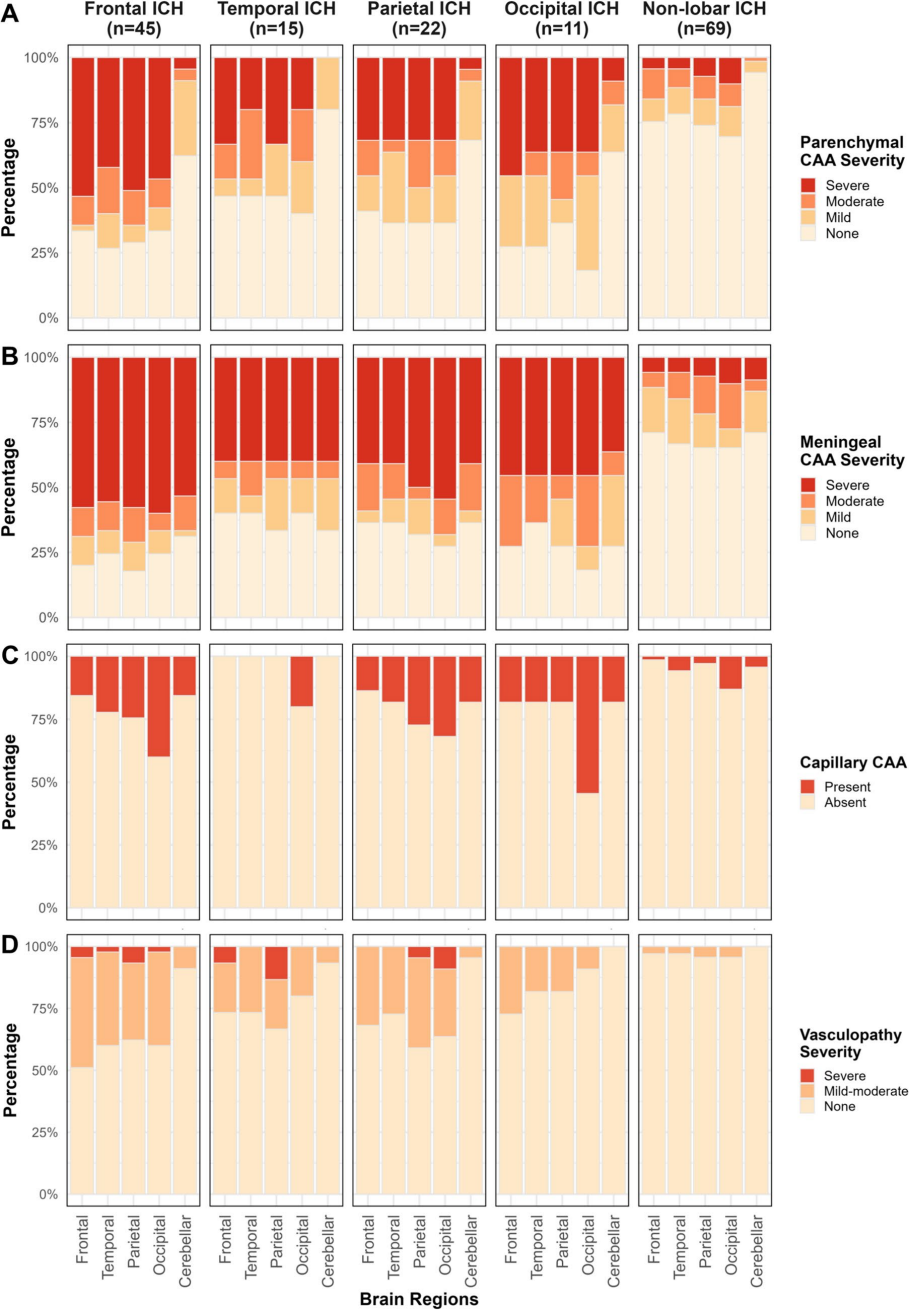
The regional distribution of CAA presence and its severity in first-ever ICH. The presence and severity of parenchymal CAA (A), meningeal CAA (B), capillary CAA (C), and CAA-associated vasculopathy (D) are shown. We assess the severity of CAA and vasculopathy pathology separately in frontal, temporal, parietal, occipital, and cerebellar regions in participants with frontal (n = 45), temporal (n = 15), parietal (n = 22), occipital (n = 11), and non-lobar ICH (n = 69). Only capillary CAA demonstrates an occipital predominance. CAA = cerebral amyloid angiopathy; ICH = intracerebral haemorrhage

All 69 participants with non-lobar ICH had moderate-to-severe non-amyloid SVD pathology, of whom 11 (15.9%) also had moderate-to-severe CAA pathology. Of 93 participants with lobar ICH, 44 (47.3%) had both moderate-to-severe CAA (which was considered sporadic in all participants) and non-amyloid SVD, 35 (37.6%) had moderate-to-severe non-amyloid SVD alone, 10 (10.8%) had moderate-to-severe CAA alone, and 4 (4.3%) had no clear underlying cause of SVD-related ICH (Fig. 2). Definite CAA, defined as moderate-to-severe parenchymal CAA with CAA-associated vasculopathy, was present in 44 (47.3%) participants with lobar ICH and in only 4 (5.8%) with non-lobar ICH. Compared to participants with non-lobar ICH, those with lobar ICH showed more severe parenchymal CAA, meningeal CAA, capillary CAA, and CAA-associated vasculopathy, and higher Thal phase for A*β* plaques and Braak stage for neurofibrillary tangles, but less non-amyloid SVD pathology (Table 2, Fig. 3). Therefore, we focused further analyses on the distribution of CAA pathology in participants with lobar ICH.

**Table 2:** Comparisons of histopathological features in participants with first-ever lobar ICH versus non-lobar ICH

	Lobar ICH (*n* = 93)	Non-lobar ICH (*n* = 69)	*P* value
Non-amyloid SVD			< 0.001
None	0	0	
Mild	14 (15.1)	0	
Moderate	27 (29.0)	6 (8.7)	
Severe	52 (55.9)	63 (91.3)	
Parenchymal CAA			< 0.001
None	26 (28.0)	46 (66.7)	
Mild	13 (14.0)	12 (17.4)	
Moderate	14 (15.1)	6 (8.7)	
Severe	40 (43.0)	5 (7.2)	
Meningeal CAA			< 0.001
None	19 (20.4)	41 (59.4)	
Mild	13 (14.0)	13 (18.8)	
Moderate	12 (12.9)	9 (13.0)	
Severe	49 (52.7)	6 (8.7)	
Capillary CAA			0.006
Absent	57 (61.3)	57 (82.6)	
Present	36 (38.7)	12 (17.4)	
CAA-associated vasculopathy			< 0.001
None	49 (52.7)	65 (94.2)	
Mild-to-moderate	40 (43.0)	4 (5.8)	
Severe	4 (4.3)	0	
Thal phase for amyloid plaques			< 0.001
0–1	26 (28.0)	41 (59.4)	
2–3	25 (26.9)	18 (26.1)	
4–5	42 (45.2)	10 (14.5)	
Braak stage for neurofibrillary tangles			0.001
0-II	44 (47.3)	53 (76.8)	
III-IV	25 (26.9)	9 (13.0)	
V-VI	24 (25.8)	7 (10.1)	

Data are shown as *n* (%) for categorical variables

*ICH* intracerebral haemorrhage, *CAA* cerebral amyloid angiopathy, *SVD* small vessel disease

### Distribution of CAA presence and its severity in lobar ICH

In lobar ICH, moderate-to-severe parenchymal CAA was observed in approximately half of participants across all cerebral lobes, but was uncommon in the cerebellum. Moderate-to-severe meningeal CAA was frequent across cerebral lobes and common in the cerebellum. Capillary CAA was most prevalent in the occipital lobe with lower frequencies in other cerebral lobes and the cerebellum. CAA-associated vasculopathy was present in roughly one-third of participants across cerebral lobes but was rare in the cerebellum (Fig. 3, Supplementary Table 2).

There were no significant differences between the four cerebral lobes in the severity of parenchymal CAA (overall GEE *P* = 0.282), meningeal CAA (overall GEE* P* = 0.298), or CAA-associated vasculopathy (overall GEE* P* = 0.068). Pairwise comparisons were shown in Supplementary Table 3. In contrast, capillary CAA showed a significant occipital predominance (overall GEE *P* < 0.001; odds ratio 2.24–3.89 for occipital versus other lobes, all FDR-adjusted *P* < 0.001). Compared with cerebral lobes, the cerebellum showed significantly lower severity of parenchymal CAA and CAA-associated vasculopathy (all FDR-adjusted *P* < 0.001), and no differences in meningeal CAA and capillary CAA except for less severe capillary CAA than in the occipital lobe (odds ratio 0.28, FDR-adjusted *P* < 0.001).

We found that there were no statistically significant differences in parenchymal CAA, meningeal CAA, capillary CAA, or CAA vasculopathy severity between ICH-affected versus ICH-unaffected hemisphere, or ICH-affected lobe versus contralateral homologous ICH-unaffected lobe (Table 3, Supplementary Fig. 2).

**Table 3:** Paired comparisons of CAA severity in ICH-affected hemisphere versus ICH-unaffected hemisphere, and in ICH-affected lobe versus contralateral homologous ICH-unaffected lobe in lobar ICH

Lobar ICH (*n* = 93)	ICH-affected hemisphere	ICH-unaffected hemisphere	*P* value	ICH-affected lobe	Contralateral ICH-unaffected lobe	*P* value
Parenchymal CAA			0.392			0.515
None	28 (30.1)	26 (28.0)		32 (34.4)	33 (35.5)	
Mild	12 (12.9)	13 (14.0)		12 (12.9)	12 (12.9)	
Moderate	13 (14.0)	16 (17.2)		11 (11.8)	13 (14.0)	
Severe	40 (43.0)	38 (40.9)		38 (40.9)	35 (37.6)	
Meningeal CAA			0.232			0.746
None	21 (22.6)	21 (22.6)		27 (29.0)	27 (29.0)	
Mild	12 (12.9)	10 (10.8)		7 (7.5)	9 (9.7)	
Moderate	11 (11.8)	15 (16.1)		13 (14.0)	11 (11.8)	
Severe	49 (52.7)	47 (50.5)		46 (49.5)	46 (49.5)	
Capillary CAA			1.00			0.625
Absent	60 (64.5)	60 (64.5)		77 (82.8)	75 (80.7)	
Present	33 (35.5)	33 (35.5)		16 (17.2)	18 (19.4)	
CAA-associated vasculopathy			0.135			0.343
None	49 (52.7)	52 (55.9)		60 (64.5)	61 (65.6)	
Mild-to-moderate	39 (41.9)	37 (39.8)		30 (32.3)	31 (33.3)	
Severe	5 (5.4)	4 (4.3)		3 (3.2)	1 (1.1)	

Data are shown as n (%) for categorical variables. Variability of distribution among paired groups is calculated using the McNemar test for 2 categories and using the Stuart-Maxwell test of marginal homogeneity across ≥ 3 categories

*CAA* cerebral amyloid angiopathy, *ICH* intracerebral haemorrhage

In prespecified subgroup analyses, all CAA and vasculopathy scores showed no differences across age groups (*P* > 0.05 for all) and were higher in carriers of *APOE* ε2 or ε4 alleles compared with non-carriers (*P* < 0.05 for all). CAA scores increased significantly with higher Thal phase and Braak stage (Cuzick’s trend test, *P* < 0.01 for all), except for capillary CAA, which did not differ across Braak stages. The distribution pattern of CAA pathology remained largely consistent across all subgroups (Supplementary Fig. 3–5).

### Diagnostic accuracy of simulated biopsy in identifying CAA presence and severity against reference standard at autopsy

We simulated a cortical biopsy in lobar ICH using a post-mortem cortical specimen from the lobe containing the ICH epicentre. For diagnosing CAA presence, Vonsattel grade ≥ 1 in the simulated biopsy specimen demonstrated a sensitivity of 100%, which did not decrease by age. The specificity for moderate-to-severe parenchymal CAA was 69.2%, ranging from 75.0% in individuals aged 56–78 years to 50.0% in those aged 86–96 years (*P* = 0.144). Similarly, the specificity for definite CAA was 55.1%, ranging from 70.6% to 41.2% across the same age groups (*P* = 0.223) (Table 4, Supplementary Table 4–6).

**Table 4:** Diagnostic accuracy of simulated cortical biopsy for identifying CAA presence in lobar ICH

Lobar ICH (*n* = 93)	Moderate-to-severe parenchymal CAA		Definite CAA	
	Vonsattel ≥ 1	Vonsattel ≥ 2	Vonsattel ≥ 1	Vonsattel ≥ 2
Sensitivity	100.0 (93.4, 100.0)	96.3 (87.3, 99.5)	100.0 (92.0, 100.0)	95.5 (84.5, 99.4)
Specificity	69.2 (52.4, 83.0)	79.5 (63.5, 90.7)	55.1 (40.2, 69.3)	63.3 (48.3, 76.6)
AUC	0.85 (0.77, 0.92)	0.88 (0.81, 0.95)	0.78 (0.71, 0.85)	0.79 (0.72, 0.87)
PPV	81.8 (70.4, 90.2)	86.7 (75.4, 94.1)	66.7 (54.0, 77.8)	70.0 (56.8, 81.2)
NPV	100.0 (87.2, 100.0)	93.9 (79.8, 99.3)	100.0 (87.2, 100.0)	93.9 (79.8, 99.3)

The Vonsattel CAA score is graded on the most advanced degree of parenchymal or meningeal CAA within the lobar specimen. Analyses are done for Vonsattel ≥ 1 or ≥ 2 in the lobe containing the ICH epicentre versus the reference standard of moderate-to-severe CAA or definite CAA (moderate-to-severe CAA plus the presence of CAA vasculopathy) at autopsy

*CAA* cerebral amyloid angiopathy, *ICH* intracerebral haemorrhage, *AUC* area under the receiver operating characteristic curve, *PPV* positive predictive value, *NPV* negative predictive value

When using a Vonsattel grade ≥ 2, sensitivity remained above 95%, while specificity was 79.5% for moderate-to-severe parenchymal CAA, varying from 87.5% in the 56–78 age group to 64.3% in the 86–96 age group (*P* = 0.312). For definite CAA, specificity was 63.3%, with a range from 82.4% to 52.9% accordingly (*P* = 0.143) (Table 4, Supplementary Table 4–6).

For evaluating CAA severity, histopathological assessment of moderate-to-severe parenchymal and meningeal CAA in the simulated biopsy demonstrated excellent specificity and good-to-excellent sensitivity compared with the reference standard. In contrast, assessment for capillary CAA and CAA-associated vasculopathy presence showed excellent specificity but poor-to-modest sensitivities, respectively (Table 5, Supplementary Table 7).

**Table 5:** Diagnostic accuracy of simulated cortical biopsy for identifying CAA and vasculopathy severity in lobar ICH

Simulated biopsy vs. reference standard	Lobar ICH (*n* = 93)
Parenchymal CAA (moderate-to-severe vs. none-to-mild)	
Sensitivity	87.0 (75.1, 94.6)
Specificity	94.9 (82.7, 99.4)
AUC	0.91 (0.85, 0.97)
PPV	95.9 (86.0, 99.5)
NPV	84.1 (69.9, 93.4)
Meningeal CAA (moderate-to-severe vs. none-to-mild)	
Sensitivity	93.4 (84.1, 98.2)
Specificity	93.8 (79.2, 99.2)
AUC	0.94 (0.88, 0.99)
PPV	96.6 (88.3, 99.6)
NPV	88.2 (72.5, 96.7)
Capillary CAA (present vs. absent)	
Sensitivity	44.4 (27.9, 61.9)
Specificity	100.0 (93.7, 100.0)
AUC	0.72 (0.64, 0.81)
PPV	100.0 (79.4, 100.0)
NPV	74.0 (62.8, 83.4)
CAA vasculopathy (present vs. absent)	
Sensitivity	75.0 (59.7, 86.8)
Specificity	100.0 (92.7, 100.0)
AUC	0.88 (0.81, 0.94)
PPV	100.0 (89.4, 100.0)
NPV	81.7 (69.6, 90.5)

Analyses are done for CAA and vasculopathy severity on the lobe containing the ICH epicentre versus the reference standard at autopsy

*CAA* cerebral amyloid angiopathy, *ICH* intracerebral haemorrhage, *AUC* area under the receiver operating characteristic curve, *PPV* positive predictive value, *NPV* negative predictive value

Restricting our analysis to the subgroup of 67 participants with lobar ICH who underwent autopsy within one year after index ICH, the sensitivity, specificity, and overall diagnostic accuracy of AUC remained consistent (Supplementary Table 8–11).

## Discussion

In this study, we characterised the distribution and severity of CAA pathology in one of the largest ICH autopsy studies, from a prospective, consecutively recruited, population-based ICH cohort. First, among participants with ICH, parenchymal CAA, meningeal CAA, and CAA-associated vasculopathy were markedly more severe in lobar ICH and were diffusely and evenly distributed across all cerebral lobes, irrespective of the ICH location. Second, for diagnosing the presence of CAA, using Vonsattel grade ≥ 1 at the post-mortem simulated biopsy as a rule-out category yielded 100% sensitivity, whereas Vonsattel grade ≥ 2 as a rule-in category achieved 79.5% specificity. These findings provide pathological insights into understanding the role of CAA severity in CAA-associated ICH and offer clinically relevant evidence for the use of cortical biopsy in the ante-mortem diagnosis of CAA.

Lobar ICH is associated with CAA pathology [4]. In our cohort, 58.1% of participants with lobar ICH had moderate-to-severe CAA (47.3% with mixed CAA and non-amyloid SVD, and only 10.8% with pure CAA). Of all lobar ICH, 37.6% had pure non-amyloid SVD and 4.3% showed no SVD-related pathological changes. This puts pure CAA in context and allows the overall prevalence of CAA and non-amyloid SVD to be ascertained from the reported frequencies. Participants who underwent autopsy were older and had a higher proportion of comorbidities than the consecutive ICH population, which likely enriched the autopsy cohort for advanced CAA and mixed SVD pathology. However, our finding was still consistent with a meta-analysis including both biopsy and autopsy studies, in which 5 studies comprising 207 participants (mean age of 73.2 years) reported a prevalence of moderate-to-severe CAA of 56.7% (41.7–71.0) in lobar ICH [8]. Another meta-analysis identified hypertension as an important risk factor for lobar ICH [28]. These findings suggest that non-amyloid SVD (particularly arteriolosclerosis) and other etiologies, may contribute to the development of lobar ICH as well. In addition, almost 16% of participants with non-lobar ICH had moderate-to-severe CAA, which was comparable to that reported in the elderly general population [8].

Evidence on whether CAA exhibits a predominantly occipital distribution remains inconsistent across studies in the general population [5, 29], AD patients [6, 30], or hospital-based ICH cohorts [7, 31]. However, few studies have further distinguished CAA pathology by vessel types or by the severity of associated vasculopathy [6]. In our population-based ICH cohort, we confirmed that the pathological scores of parenchymal CAA, meningeal CAA, and CAA-associated vasculopathy were similar across cerebral lobes. These corresponded to CAA type II, which involved larger arteries or arterioles in the cortex and was more closely associated with vessel rupture and subsequent ICH [32]. Furthermore, we did not observe differences in CAA severity or CAA-associated vasculopathy between ICH-affected and unaffected regions. Our findings indicate that type II CAA was diffusely distributed even in participants with focal ICH. This observation has two implications. First, it may strengthen the hypothesis that CAA is derived from a generalized failure of A*β* clearance and is exacerbated by widespread morphological and functional changes of the vascular wall [33]. Second, this seems to challenge the previous understanding that CAA-ICH is driven primarily by locally more severe CAA or vasculopathy [11, 12]. Instead, in this work, CAA severity and vasculopathy appear diffuse, suggesting network-wide rather than local effects whereby CAA progression may place the lobar cerebral microvasculature in a ‘critical state’. Additional triggers, including inflammatory processes, hemodynamic fluctuations, coexisting non-amyloid SVD, vascular risk factors, including hypertension, or anticoagulant medications, are likely required to precipitate vessel rupture at susceptible or random sites [34–36]. It should be noted, however, that haemorrhagic lesions are thought to occur during late stages of CAA disease progression and the observed CAA distribution might therefore have reached a plateau. As such, studies focused on assessing CAA pathology and distribution in earlier stages of disease progression remains to be explored [37].

In contrast, capillary CAA (CAA type I) showed an occipital predominance, which was more often linked to cerebral microbleeds but generally not with ICH [32, 38]. This pattern remained consistent across age, different severity of AD pathology, and *APOE* status groups. One potential explanation relates to structural and functional differences between the anterior and posterior circulation. Occipital vessels were anatomically thicker with more collagen IV in the basement membrane compared with vessels in other lobes [39], which reduced A*β* clearance within perivascular pathways, thereby promoting capillary A*β* accumulation. Furthermore, posterior watershed regions, located at the distal border zones between major arterial territories are particularly vulnerable to hypoperfusion and vasculopathic changes [40, 41], and may play a contributory role.

Cerebellar CAA and its association with superficial cerebellar ICH have been increasingly reported [31, 42, 43]. In our cohort, 7 participants presented with cerebellar ICH, of whom only one had a pathological diagnosis of CAA. Moderate-to-severe parenchymal CAA and CAA-associated vasculopathy were all less frequent in the cerebellum (< 15%) compared with cerebral lobes, whereas meningeal CAA occurred at a similar frequency of 60%, consistent with a previous study in AD [44]. It may be attributed to the unique anatomic architecture of the cerebellum, including shorter penetrating arterioles, a greater contribution of meningeal vessels to cortical blood supply, and perhaps enhanced A*β* drainage via the meningeal lymphatic system [45].

Recent findings from the ENRICH trial demonstrate improved functional outcomes with minimally invasive hematoma evacuation, particularly in lobar ICH [46]. It is anticipated that this surgical approach may facilitate obtaining cortical specimens to identify the etiology of ICH in a greater number of participants. Until now, only one study has assessed the diagnostic accuracy of cortical biopsies for CAA, including 28 post-mortem specimens from only two participants with CAA-ICH and without non-CAA ICH controls [9]. In our study, the CAA pathological score in the cerebral lobe containing the ICH epicentre was used as a simulated biopsy. Applying Vonsattel grade ≥ 1 as the rule-out category achieved 100% sensitivity, and Vonsattel grade ≥ 2 as the rule-in category had an overall specificity of 79.5%. The specificity was numerically lower but not statistically significant with advancing age, from 88.0% in participants younger than 85 years to 64.3% in those aged 85 years or older. These standardised diagnostic performance metrics for cortical biopsy–based CAA diagnosis cannot be directly extrapolated to in vivo biopsy, which typically involves smaller tissue volumes and greater sampling uncertainty. These findings highlight that pathological diagnosis from biopsy specimens, especially in elderly adults, requires careful interpretation, emphasizing the need for integrated clinical and neuroimaging assessment to avoid false-positive diagnoses.

Currently, three approaches are employed for the ante-mortem diagnosis of CAA-related ICH: the Boston criteria version 2.0 based on MRI, the Edinburgh CT and genetic criteria, and cortical biopsy or haematoma evacuation [18, 27]. Both the Boston and Edinburgh criteria demonstrated excellent sensitivity for the rule-out category. For the rule-in category, subarachnoid haemorrhageand either *APOE* ε4 possession or finger-like projections in the Edinburgh criteria achieved 96% specificity and probable CAA in the Boston criteria had 92.9% specificity against autopsy. Since the Edinburgh criteria were derived from the same cohort (62/95 participants), and only 17 participants in our study had MRI, direct head-to-head comparison of biopsy with either the Edinburgh or Boston criteria was not feasible. In addition, our study differed from the Boston criteria study in both pathological reference standard (moderate-to-severe parenchymal CAA versus Vonsattel ≥ 2) and study population (lobar ICH only versus both lobar and non-lobar ICH) [27], and, therefore, direct numerical comparison of sensitivity and specificity would not be meaningful. In selected clinical scenarios, biopsy-based diagnosis for CAA may provide additional value, including indeterminate haemorrhagic morphology on CT, MRI unavailability or contraindication, the presence of both deep and lobar ICHs or microbleeds, or the need to exclude other causes for ICH. In contrast, in older patients with clear imaging-based diagnostic classifications, for whom the specificity of biopsy is relatively low, cortical biopsy is unlikely to provide additional diagnostic information.

The strengths of this study include being the first consecutive population-based cohort with comprehensive and standardised pathological assessment to investigate CAA and non-amyloid SVD pathology in ICH, and minimizing selection and information biases through prospective recruitment and blinded assessments.

The study has several limitations. First, although we tried to limit selection bias, participants who underwent autopsy were older and had more frequent pre-ICH dementia (Supplementary Table 1), both of which are associated with more advanced CAA pathology [9, 47], potentially leading to an overestimation of CAA severity. Second, the pathological severity of non-amyloid SVD was assessed globally and only in the left hemisphere. Although the pathological severity of non-amyloid SVD is generally considered symmetrical, some degree of hemispheric asymmetry may exist, especially in unilateral ICH. In addition, non-amyloid SVD was not assessed by individual brain regions, so we were unable to explore its regional distribution in relation to ICH lesions. Given that nearly half of the participants with lobar ICH in our cohort had both moderate-to-severe CAA and non-amyloid SVD, investigating their distributions concurrently might help clarify the respective contributions of CAA and non-amyloid SVD to ICH. Third, simulated cortical biopsies were based on the post-mortem specimen from the ICH-affected lobe. As they were obtained from the same brain and possibly the same block as the reference standard, this could introduce potential incorporation bias. Moreover, the routinely sampled cortical region might not accurately replicate peri-hematoma sampling and likely provides more tissue than a real biopsy. These factors may have overestimated the diagnostic sensitivity of the cortical biopsy. Fourth, due to the disruptive nature of ICH, it is very challenging to assess the pathological severity of the blood vessels that have ruptured, and some pathological features of these vessels might have been missed. Fifth, the interval between index ICH and autopsy was prolonged for some participants; 28% participants had autopsy done more than one year after the index ICH, which could allow CAA severity to increase over time. However, sensitivity analyses restricted to those with an autopsy within one year showed no reduction in the diagnostic performance. Sixth, our autopsy cohort enrolled almost exclusively White participants of European ancestry from a single geographic region, highlighting the need to assess the generalisability of our findings in other racial and ethnic populations. Finally, due to the ICH-based cohort design, we were unable to include participants with non-ICH CAA. Future studies comparing CAA pathology between ICH and non-ICH CAA subtypes may further elucidate the relationship between CAA and ICH.

In conclusion, our autopsy-based analysis reveals that cerebral CAA pathology in SVD-related ICH is diffusely distributed rather than confined to focal haemorrhagic regions. There is still a need to better understand the factors that trigger bleeding in vessels affected by CAA. Additionally, cortical biopsy offers excellent sensitivity but only modest-to-good specificity for the ante-mortem diagnosis of CAA-related ICH versus a reference standard at autopsy, particularly in the elderly. This highlights the careful interpretation of biopsy results in clinical decision-making by integrating imaging and clinical information to optimise diagnostic reliability and inform management strategies.

## Supplementary Information

Below is the link to the electronic supplementary material.

Supplementary file1 (DOCX 89 KB)

Supplementary file2 (DOCX 2949 KB)

## Data Availability

The data that support the findings of this study are available from the corresponding author upon reasonable request.
