# Supplementary material for: Pathological insights into cerebral amyloid angiopathy underlying intracerebral haemorrhage: population-based autopsy study
Source: Acta Neuropathol. 2026 Jan 24;151(1):8. doi: 10.1007/s00401-026-02980-0 (PMC12831695; doi:10.1007/s00401-026-02980-0)
Supplement: Supplementary file 1 — Supplementary file1 (DOCX 89 KB) [file 401_2026_2980_MOESM1_ESM.docx]

**Supplementary Material 1: Systematic review of autopsy-based studies on cerebral amyloid angiopathy pathology with intracerebral haemorrhage**

**Pathological insights into cerebral amyloid angiopathy underlying intracerebral haemorrhage: population-based autopsy study**

We did a systematic review of studies on the presence, severity, or distribution of cerebral amyloid angiopathy (CAA) pathology at autopsy in individuals with intracerebral haemorrhage (ICH) published in MEDLINE (from 1946 to Aug 1, 2025) and Embase (from 1974 to Aug 1, 2025) using comprehensive electronic search strategies combining terms for “(brain$ or cerebr$ or intracerebr$) AND (h?emorrhag$ or h?ematoma$)” and “(amyloid angiopath$ or vascular amyloidosis or amyloid beta or congo?red)” and “(patholog$ or post?mortem$ or autops$ or necrops$)”. We included 29 case series that enrolled >5 individuals with ICH, provided a clear histopathological definition for the presence of CAA, and described the prevalence, severity, or distribution of CAA at autopsy.

In summary, the main findings are (details in **Table**):

1. CAA pathology has been investigated in participants with ICH from hospital-based ICH cohorts, Alzheimer’s disease cohorts, and population-based autopsy series (e.g. ~30 participants with ICH among 1,000 elderly individuals), but not in population-based consecutive ICH cohorts.
2. A variety of grading and staging systems have been proposed to evaluate CAA pathology in ICH. Many of these rely primarily on the proportion or number of affected vessels within a cortical region, which limits their capacity to capture the global CAA pathology of different vessel types and CAA vasculopathy. Among them, the Vonsattel and Love scales are the most widely applied for grading both CAA severity and associated vasculopathy across different brain regions.
3. CAA is associated with lobar ICH. A few studies have confirmed the association of *APOE* ε2 or ε4 alleles with CAA-associated ICH and the comorbidity of arteriolosclerosis and CAA in ICH. However, it remains uncertain whether CAA associated with ICH exhibits a predominantly occipital distribution. Moreover, no study has directly examined whether CAA-associated ICH arises from more severe CAA pathology or vasculopathy within the ICH-affected region compared with other brain regions.
4. Only one study has assessed the diagnostic accuracy of cortical biopsy for CAA, based on 28 simulated specimens from only two participants with CAA-associated ICH and without controls of non-CAA ICH. In that study, specificity was calculated as 100% minus the probability of encountering the same degree of CAA in brain tissue from the general elderly population.

**Table. Design, pathological methods, and main findings of studies of CAA pathology with ICH at autopsy-based studies.** Studies are in ascending chronological order by year of publication.

| **Study** | **Study design** | **Cases** | **Staining** | **Definition of CAA presence** | **CAA rating method** | **Vessel types** | **Brain regions** | **Main findings about CAA pathology with ICH** |
| --- | --- | --- | --- | --- | --- | --- | --- | --- |
| **Gilles, 1984^1^** | Hospital-based autopsies, Belgium | 6 CAA-ICH, mean age 69.5y | Congo red | Moderate-to-severe CAA | Number of CAA-involved vessels (0–3) | LM & IP | Cerebral lobes, BG, brainstem, cerebellum | CAA: mainly in meninges and cortex, but rare in white matter and deep regions. |
| **Ishii, 1984^2^** | Hospital-based autopsies, Japan | 60 ICH, from 1080 autopsies | Congo red | Moderate-to-severe CAA | None, mild, moderate, and severe (0-3) | LM & IP | Cerebral lobes, hippocampus, BG, brainstem, cerebellum, haematoma | CAA prevalence: 11.7% (7/60) of ICH.  CAA: mainly in meninges and cortex, involving media and adventitia, sparing endothelium within vessel wall. |
| **Cosgrove, 1985^3^** | Hospital-based autopsies, Canada | 16 CAA-ICH from 24 autopsies of CAA, mean age 80y | Congo red | Amyloid+ vessels in ≥1 brain area | CAA-involved vessels / total vessels (0–3), and number of involved lobes (0-3) | LM & IP | Cerebral lobes, hippocampus, BG, midbrain, cerebellum | CAA severity: more extensive in CAA-ICH than non-ICH CAA.  ICH lesions: all in cortical and subcortical regions, most with SAH.  CAA Distribution: frontal > occipital > parietal > temporal > BG > cerebellum/brainstem. |
| **Masuda, 1988^4^** | Community-based autopsies, Japan | 26 ICH (23 deep, 2 lobar, 1 cerebellar), from 400 autopsies | Congo red | Amyloid+ vessels ≥1% | CAA-involved vessels / total vessels (0–4) | IP | Cerebral lobes, hippocampus, BG | CAA prevalence: 11.5% (3/26) of ICH.  Only 1 cerebellar ICH was due to CAA. |
| **Vonsattel, 1991^5^** | Hospital-based autopsies, USA | 17 CAA-ICH (15 autopsies, 2 biopsies, mean age 73.7y) vs. 66 general hospital patients aged >75y, 70 neuropsychiatric patients | Congo red | ≥1 amyloid+ vessel | Severity of amyloid deposition and vasculopathy (0-3) | LM & IP | Cerebral lobes, hippocampus, BG, cerebellum, brainstem | CAA prevalence: 100% of CAA-ICH, 45% (30/66) of general hospital patients, 54% (38/70) of neuropsychiatric patients.  CAA distribution: frontal > parietal/occipital > temporal lobes.  CAA severity: more severe in ICH; fibrinoid necrosis observed only in ICH (12/17); microaneurysm observed only in severe CAA. |
| **Ishihara, 1991^6^** | Hospital-based autopsies, Japan | 50 ICH (mean age 72y) vs. 56 cerebral infarction, 63 controls | Congo red and IHC | A few amyloid+ vessels after extensive search | Number of CAA-involved vessels (0–3) | NA | Cerebral lobes, central grey matter, brainstem, cerebellum | CAA prevalence: 75% (6/8) of pure lobar ICH, 27% (10/37) of pure deep ICH, 25% (14/56) of cerebral infarction, and 32% (20/63) of controls. |
| **Ng, 1991^7^** | Hospital-based autopsies, China | 49 ICH vs. 210 randomly selected patients over 40y | Congo red | ≥1 amyloid+ vessel | Vinters scale^8^: number of CAA-involved vessels (0–3) | LM & IP | Hematoma | CAA prevalence: 10% (21/210) of controls and 8.2% (4/49) of ICH. |
| **Yoshimura, 1992^9^** | Hospital-based autopsies, Japan | 150 ICH, from 1700 autopsies, mean age 78.6y | Congo red and IHC | ≥1 amyloid+ vessel | NA | NA | Cerebral cortex, BG, thalamus, brainstem, cerebellum, haematoma | CAA prevalence: 8.0% (12/150) of ICH, 21.1% (8/38) of lobar ICH.  CAA distribution: occipital (39%) > temporal (28%) > frontal (18%) > parietal (15%) lobes.  CAA vasculopathy: widespread in meningo-cortical regions of cerebrum and cerebellum. |
| **Yoshinori and Yamada, 1993^10, 11^** | Hospital-based autopsies, Japan | 101 ICH, from 1000 autopsies, mean age 82.9y | Congo red and IHC | ≥4/10 amyloid+ vessels and no other lesions | CAA-involved vessels / total vessels | LM & IP | Cerebral lobes, cerebellum | CAA prevalence: 10.9% of ICH (31% [9/29] of lobar ICH and 14.3% [2/14] of cerebellar ICH); with AS in 27.3% of CAA-ICH.  CAA distribution: temporal > frontal > occipital lobes.  CAA severity: moderate-to-severe CAA in LM and IP vessels.  All ICH lesions with SAH. |
| **Iwamoto, 1993^12^** | NA, Japan | 17 ICH vs. 11 controls, mean age 76y | Congo red | Partial amyloid+ deposition | CAA-involved vessels / total vessels, and degree of amyloid deposition (0-3) | LM & IP | Cerebral lobes, haematoma | CAA prevalence and severity: higher in lobar ICH (52.2%) than in deep ICH (12.2%) and non-ICH group (17.9%). |
| **Greenberg, 1995^13^** | Hospital-based autopsies, USA | 15 CAA-ICH (5 autopsies, 10 biopsies, 7 clinical diagnosis, mean age 77.1y) vs. 93 CAA autopsies | Congo red | Severe CAA, no other ICH cause. | Vonsattel scale (0-3)^5^ | LM & IP | Cerebral lobes, BG, cerebellum, brainstem | CAA-ICH (n=15): higher *APOE* ε4 frequency of 0.40 vs 0.14 in controls, consistent after adjusting for AD pathology. |
| **Nicoll, 1997^14^** | Multicentre, hospital-based autopsies, UK and USA | 36 CAA-ICH (33 autopsies and 3 biopsies, mean age 72y) vs. 61 AD and 43 elder controls | IHC | Abundant amyloid+ vessels, no other ICH cause. | NA | NA | NA | CAA-ICH: *APOE* ε2 frequency of 0.25, *APOE* ε4 of 0.18.  *APOE* ε4: higher in CAA-ICH with AD than those without (17 vs. 13); *APOE* ε2: higher in CAA-ICH regardless of AD than controls. |
| **Greenberg, 1997^15^** | Hospital-based autopsies, USA | 7 CAA (2 mild CAA, 3 moderate-to-severe CAA without ICH, and 2 CAA-ICH) vs. 784 non-ICH autopsies | Congo red and IHC | Severe CAA and corticosubcortical ICH, no other ICH cause. | Vonsattel scale (0-4)^15^ | LM & IP | Cerebral lobes, cerebellum | CAA severity: more severe in LM than IP vessels.  CAA distribution: occipital predominance in LM, not IP vessels.  Diagnostic accuracy of biopsy ^#^: Vonsattel ≥1, 100% sensitivity, 95%-77% specificity with advancing age; Vonsattel ≥2: 93% sensitivity, 98%-88% specificity.  Fibrinoid necrosis: 46.4% (13/28) in CAA-ICH, 0% in mild CAA, 9.5% (4/42) in moderate-to-severe CAA without ICH. |
| **Fazekas, 1999^16^** | Hospital-based autopsies, Austria | 11 ICH (7 lobar, 4 deep), mean age 72.2y | Congo red | Moderate-to-severe CAA | Vonsattel scale (0-3)^5^ | NA | NA | Prevalence of CAA with vasculopathy: 18.2 % (2/11) of ICH. |
| **Knudsen, 2001^17^** | Hospital-based autopsies, USA | 14 lobar ICH diagnosed with possible or probable CAA on the Boston criteria, mean age ~74y | Congo red | Complete amyloid replacement, vessel wall cracking, ≥1 paravascular haemorrhage focus, no other ICH cause. | NA | NA | NA | CAA prevalence: 100% (6/6) of probable CAA and 50% (4/8) of possible CAA. |
| **Xu, 2003^18^** | Hospital-based autopsies, China | 65 ICH, from 362 autopsies of elderly adults | Congo red and IHC | ≥1 amyloid+ vessel | Number of CAA-involved vessels and number of involved lobes (0–3) | LM & IP | Cerebral lobes, subcortical white matter, cerebellum | CAA prevalence: 32.3% (21/65) of ICH, 50% (2/4) of cerebellar ICH.  CAA was the cause for 16.9% (11/65) of ICH. |
| **Oide, 2003^19^** | Hospital-based autopsies, Japan | 12 lobar ICH with proven CAA | Congo red and IHC | A few amyloid+ vessels after extensive search | Number of CAA-involved vessels (0–3) | LM & IP | Cortical regions including the grey and white matter, away from hematoma | CAA prevalence: 100% (12/12) of grey matter and 8.3% (1/12) of white matter. Senile plaques: 100% (12/12); neurofibrillary tangles: 50% (6/12);  AS: 16.7% (2/12). |
| **Takeda, 2003^20^** | Hospital-based autopsies, Japan | 6 ICH with proven CAA, mean age 77.3y | IHC | Amyloid+ vessel | Number of CAA-involved vessels | LM & IP | Cortex around haematoma | CAA prevalence and severity: more extensive in LM than IP vessels.  All CAA-ICH had multiple SAHs. |
| **Ritter, 2005^21^** | Hospital-based autopsies, Hungary | 64 ICH (24 lobar, 40 deep, mean age 69.3y) vs. 65 without ICH; all with HTN | Congo red | Congo-red-positive in the vessel wall | Vonsattel scale (0-3)^5^ | LM & IP | Cerebral lobes, BG, cerebellum, haematoma | CAA prevalence: 23% (15/64) of ICH and 8% (5/65) of controls.  CAA was associated with lobar ICH.  CAA severity: similar in ICH vs. non-ICH.  CAA distribution: mainly in occipital and frontoparietal lobes; absent in BG, less in cerebellum (4/15). |
| **Jellinger, 2007^22^** | Hospital-based autopsies, Austria | 60 ICH, from 1100 autopsies of elderly, mean age 79.1y | IHC | ≥ scattered amyloid+ in vessels | Olichney scale (0-4)^23^ | LM & IP | Frontal and occipital lobes, hippocampus | CAA prevalence: 50.6% (526/1041) without ICH and 42.4% (25/59) with ICH.  CAA severity: severe CAA in 20.2% (210/1041) without ICH and 33.9% (20/59) with ICH.  CAA distribution: occipital > frontal > parietal > temporal lobes. |
| **Attems, 2008^24^** | Hospital-based autopsies, Austria | 115 ICH, from 2060 autopsies, mean age ~81y | IHC | ≥ scattered amyloid+ in vessels | Olichney scale^23^ | LM & IP | Frontal and occipital lobes, hippocampus | CAA prevalence: 49% (953/1945) without ICH, in 48.7% (56/115) with ICH.  CAA severity: severe CAA in 30.9% (290/953) without ICH, 80.4% (45/56) with ICH. |
| **Mendel, 2013^25, 26^** | Hospital-based autopsies, Poland | 189 ICH, mean age 79.9y | Congo red and IHC | ≥1 amyloid+ vessel | Vonsattel and Mountjoy scales^15, 27^ | LM & IP, including vein | NA | CAA prevalence: 22% (42/189) of ICH.  CAA severity: Vonsattel scale, 10% mild, 14% moderate, 76% severe; Mountjoy scale, 5% grade 3, 95% grade 4.  AS: 14% grade 1, 49% grade 2, 20% grade 3, 17% grade 4.  78% (33/42) of CAA-ICH had amyloid+ veins. |
| **Charidimou, 2015^28^** | Hospital-based autopsies/biopsies, USA | 105 CAA: 52 autopsies, 53 biopsies; 54 ICH, 51 non-ICH, mean age 72.7y | Congo red and IHC | ≥1 amyloid+ vessel | Vonsattel scale (0-4)^15^ | LM & IP, including vein | NA | CAA severity and vasculopathy: similar in CAA-ICH vs. non-ICH CAA.  Neuritic plaques without tangles: more frequent in CAA-ICH; neurofibrillary tangles were more frequent in non-ICH CAA.  *APOE* ε2: 48.7% in CAA-ICH vs. 21.4% in non-ICH CAA (*P* = 0.075). *APOE* ε4: 53.9% in CAA-ICH vs. 85.7% in non-ICH CAA (*P* = 0.035). |
| **De Reuck, 2016^29^** | Hospital-based autopsies, France | 30 lobar ICH, from 252 autopsies, mean age 75.7y | IHC | Majority of amyloid+ vessels in ≥3/4 examined samples | NA | NA | Cerebral lobes, hippocampus | CAA prevalence: 56.7% (17/30) of lobar ICH.  CAA distribution: mainly parieto-occipital.  CAA-ICH had more severe white matter changes, cortical microinfarcts, and microbleeds than non-CAA ICH. |
| **Lauer, 2016^30^** | Hospital-based autopsies, USA | 13 CAA-ICH, from 49 autopsies of CAA, mean age ~75y | Congo red and IHC | Vonsattel grade ≥2 | Vonsattel scale (0-4)^15^ | NA | NA | Presence of microinfarcts was associated with the presence of ICH in CAA. |
| **Guidoux, 2018^31^** | Hospital-based autopsies, France | 81 ICH (51 deep, 30 lobar, median age 74y) vs. 59 controls | Congo red and IHC | Consus using qualitative quotation | NA | NA | Cerebral lobes, cerebellum | CAA prevalence: 24.7% (20/81) of ICH, 50.0% (15/30) of lobar ICH, 9.8% (5/51) of deep ICH.  HTN frequently coexists with CAA in ICH. |
| **Rodrigues, 2018^32^** | Community-based autopsies, UK | 110 first-ever ICH (62 lobar, 48 non-lobar), median age 83y ***** | IHC | Moderate-to-severe parenchymal CAA | Love scale^33^ | LM & IP, including capillary | Cerebral lobes, cerebellum | CAA prevalence: 58% (36/62) of lobar ICH and 12.5% (6/48) of non-lobar ICH.  Development and internal validation of the Edinburgh CT and genetic criteria for CAA-ICH. |
| **Charidimou, 2022^34^** | Hospital-based autopsies, North America and Europe | 75 ICH, median age ~70y | IHC | Vonsattel grade ≥2 | Vonsattel scale (0-4)^15^ | NA | NA | The derivation of the Boston criteria v2.0 for CAA. |
| **Jolink, 2023^35^** | Hospital-based autopsies, Netherlands | 20 ICH, mean age at death 77y | Congo red and IHC | Vonsattel grade ≥2 | Vonsattel scale (0-4)^15^ | IP, including capillary | Cortex | Lobar ICH (n=9): 6 pure CAA, 1 hereditary CAA, 1 both CAA and other SVD, 1 neither.  Non-lobar ICH (n=11): 5 pure other SVD, 2 both CAA and other SVD, 4 other causes.  Microinfarcts and microbleeds: more often in superficial cortex of lobar ICH and in deep cortex of non-lobar ICH.  CAA severity: a trend toward more severe CAA in lobar ICH; other pathology features similar. |

**^#^**In the study by Greenberg et al, sensitivity was calculated as the likelihood of finding a given degree of CAA in specimens taken from brain with CAA-related haemorrhage, and specificity was calculated as 100% minus the percent likelihood of encountering that degree of CAA in a brain sample from the general elderly population.^15^

*Of the 162 participants in our study, 110 overlapped with this cohort.

CAA, cerebral amyloid angiopathy; ICH, intracerebral haemorrhage; IHC, immunohistochemical staining; LM, leptomeningeal; IP, intraparenchymal; amyloid+, amyloid positive; BG, basal ganglia; NA, not available; SAH, subarachnoid haemorrhage; AS, arteriolosclerosis; HTN, hypertension.

**References**

1. Gilles C, Brucher JM, Khoubesserian P, Vanderhaeghen JJ. Cerebral amyloid angiopathy as a cause of multiple intracerebral hemorrhages. *Neurology*. 1984;34(6):730-735. doi:10.1212/wnl.34.6.730

2. Ishii N, Nishihara Y, Horie A. Amyloid angiopathy and lobar cerebral haemorrhage. *J Neurol Neurosurg Psychiatry*. 1984;47(11):1203-1210. doi:10.1136/jnnp.47.11.1203

3. Cosgrove GR, Leblanc R, Meagher-Villemure K, Ethier R. Cerebral amyloid angiopathy. *Neurology*. 1985;35(5):625-631. doi:10.1212/wnl.35.5.625

4. Masuda J, Tanaka K, Ueda K, Omae T. Autopsy study of incidence and distribution of cerebral amyloid angiopathy in hisayama, japan. *Stroke*. 1988;19(2):205-210. doi:10.1161/01.str.19.2.205

5. Vonsattel JP, Myers RH, Hedley-Whyte ET, et al. Cerebral amyloid angiopathy without and with cerebral hemorrhages: A comparative histological study. *Ann Neurol*. 1991;30(5):637-649. doi:10.1002/ana.410300503

6. Ishihara T, Takahashi M, Yokota T, et al. The significance of cerebrovascular amyloid in the aetiology of superficial (lobar) cerebral haemorrhage and its incidence in the elderly population. *J Pathol*. 1991;165(3):229-234. doi:10.1002/path.1711650306

7. Ng TH, Leung SY, Wong MP. Cerebral amyloid angiopathy in chinese: Incidence and significance. *Clin Neurol Neurosurg*. 1991;93(1):19-23. doi:10.1016/0303-8467(91)90004-9

8. Vinters HV, Gilbert JJ. Cerebral amyloid angiopathy: Incidence and complications in the aging brain. Ii. The distribution of amyloid vascular changes. *Stroke*. 1983;14(6):924-928. doi:10.1161/01.str.14.6.924

9. Yoshimura M, Yamanouchi H, Kuzuhara S, et al. Dementia in cerebral amyloid angiopathy: A clinicopathological study. *J Neurol*. 1992;239(8):441-450. doi:10.1007/BF00856809

10. Itoh Y, Yamada M, Hayakawa M, Otomo E, Miyatake T. Cerebral amyloid angiopathy: A significant cause of cerebellar as well as lobar cerebral hemorrhage in the elderly. *J Neurol Sci*. 1993;116(2):135-141. doi:10.1016/0022-510x(93)90317-r

11. Yamada M, Itoh Y, Otomo E, Hayakawa M, Miyatake T. Subarachnoid haemorrhage in the elderly: A necropsy study of the association with cerebral amyloid angiopathy. *J Neurol Neurosurg Psychiatry*. 1993;56(5):543-547. doi:10.1136/jnnp.56.5.543

12. Iwamoto N, Ishihara T, Ito H, Uchino F. Morphological evaluation of amyloid-laden arteries in leptomeninges, cortices and subcortices in cerebral amyloid angiopathy with subcortical hemorrhage. *Acta Neuropathol*. 1993;86(5):418-421. doi:10.1007/BF00228574

13. Greenberg SM, Rebeck GW, Vonsattel JP, Gomez-Isla T, Hyman BT. Apolipoprotein e epsilon 4 and cerebral hemorrhage associated with amyloid angiopathy. *Ann Neurol*. 1995;38(2):254-259. doi:10.1002/ana.410380219

14. Nicoll JA, Burnett C, Love S, et al. High frequency of apolipoprotein e epsilon 2 allele in hemorrhage due to cerebral amyloid angiopathy. *Ann Neurol*. 1997;41(6):716-721. doi:10.1002/ana.410410607

15. Greenberg SM, Vonsattel JP. Diagnosis of cerebral amyloid angiopathy. Sensitivity and specificity of cortical biopsy. *Stroke*. 1997;28(7):1418-1422. doi:10.1161/01.str.28.7.1418

16. Fazekas F, Kleinert R, Roob G, et al. Histopathologic analysis of foci of signal loss on gradient-echo t2*-weighted mr images in patients with spontaneous intracerebral hemorrhage: Evidence of microangiopathy-related microbleeds. *AJNR Am J Neuroradiol*. 1999;20(4):637-642.

17. Knudsen KA, Rosand J, Karluk D, Greenberg SM. Clinical diagnosis of cerebral amyloid angiopathy: Validation of the boston criteria. *Neurology*. 2001;56(4):537-539. doi:10.1212/wnl.56.4.537

18. Xu D, Yang C, Wang L. Cerebral amyloid angiopathy in aged chinese: A clinico-neuropathological study. *Acta Neuropathol*. 2003;106(1):89-91. doi:10.1007/s00401-003-0706-1

19. Oide T, Takahashi H, Yutani C, Ishihara T, Ikeda S. Relationship between lobar intracerebral hemorrhage and leukoencephalopathy associated with cerebral amyloid angiopathy: Clinicopathological study of 64 japanese patients. *Amyloid*. 2003;10(3):136-143. doi:10.3109/13506120308998994

20. Takeda S, Yamazaki K, Miyakawa T, et al. Subcortical hematoma caused by cerebral amyloid angiopathy: Does the first evidence of hemorrhage occur in the subarachnoid space? *Neuropathology*. 2003;23(4):254-261. doi:10.1046/j.1440-1789.2003.00506.x

21. Ritter MA, Droste DW, Hegedus K, et al. Role of cerebral amyloid angiopathy in intracerebral hemorrhage in hypertensive patients. *Neurology*. 2005;64(7):1233-1237. doi:10.1212/01.WNL.0000156522.93403.C3

22. Jellinger KA, Lauda F, Attems J. Sporadic cerebral amyloid angiopathy is not a frequent cause of spontaneous brain hemorrhage. *Eur J Neurol*. 2007;14(8):923-928. doi:10.1111/j.1468-1331.2007.01880.x

23. Olichney JM, Hansen LA, Hofstetter CR, et al. Cerebral infarction in alzheimer's disease is associated with severe amyloid angiopathy and hypertension. *Arch Neurol*. 1995;52(7):702-708. doi:10.1001/archneur.1995.00540310076019

24. Attems J, Lauda F, Jellinger KA. Unexpectedly low prevalence of intracerebral hemorrhages in sporadic cerebral amyloid angiopathy: An autopsy study. *J Neurol*. 2008;255(1):70-76. doi:10.1007/s00415-008-0674-4

25. Mendel TA, Wierzba-Bobrowicz T, Stepien T, Szpak GM. The association between cerebral amyloid angiopathy and atherosclerosis in patients with intracerebral hemorrhages. *Folia Neuropathol*. 2013;51(3):243-249. doi:10.5114/fn.2013.37709

26. Mendel T, Wierzba-Bobrowicz T, Stepien T, Szpak GM. Beta-amyloid deposits in veins in patients with cerebral amyloid angiopathy and intracerebral haemorrhage. *Folia Neuropathol*. 2013;51(2):120-126. doi:10.5114/fn.2013.35954

27. Mountjoy CQ, Tomlinson BE, Gibson PH. Amyloid and senile plaques and cerebral blood vessels. A semi-quantitative investigation of a possible relationship. *J Neurol Sci*. 1982;57(1):89-103. doi:10.1016/0022-510x(82)90113-7

28. Charidimou A, Martinez-Ramirez S, Shoamanesh A, et al. Cerebral amyloid angiopathy with and without hemorrhage: Evidence for different disease phenotypes. *Neurology*. 2015;84(12):1206-1212. doi:10.1212/WNL.0000000000001398

29. De Reuck J, Cordonnier C, Deramecourt V, et al. Lobar intracerebral haematomas: Neuropathological and 7.0-tesla magnetic resonance imaging evaluation. *J Neurol Sci*. 2016;369121-125. doi:10.1016/j.jns.2016.08.012

30. Lauer A, van Veluw SJ, William CM, et al. Microbleeds on mri are associated with microinfarcts on autopsy in cerebral amyloid angiopathy. *Neurology*. 2016;87(14):1488-1492. doi:10.1212/WNL.0000000000003184

31. Guidoux C, Hauw JJ, Klein IF, et al. Amyloid angiopathy in brain hemorrhage: A postmortem neuropathological-magnetic resonance imaging study. *Cerebrovasc Dis*. 2018;45(3-4):124-131. doi:10.1159/000486554

32. Rodrigues MA, Samarasekera N, Lerpiniere C, et al. The edinburgh ct and genetic diagnostic criteria for lobar intracerebral haemorrhage associated with cerebral amyloid angiopathy: Model development and diagnostic test accuracy study. *Lancet Neurol*. 2018;17(3):232-240. doi:10.1016/S1474-4422(18)30006-1

33. Love S, Chalmers K, Ince P, et al. Development, appraisal, validation and implementation of a consensus protocol for the assessment of cerebral amyloid angiopathy in post-mortem brain tissue. *Am J Neurodegener Dis*. 2014;3(1):19-32.

34. Charidimou A, Boulouis G, Frosch MP, et al. The boston criteria version 2.0 for cerebral amyloid angiopathy: A multicentre, retrospective, mri-neuropathology diagnostic accuracy study. *Lancet Neurol*. 2022;21(8):714-725. doi:10.1016/S1474-4422(22)00208-3

35. Jolink WMT, van Veluw SJ, Zwanenburg JJM, et al. Histopathology of cerebral microinfarcts and microbleeds in spontaneous intracerebral hemorrhage. *Transl Stroke Res*. 2023;14(2):174-184. doi:10.1007/s12975-022-01016-5
