# Supplementary material for: Pathological insights into cerebral amyloid angiopathy underlying intracerebral haemorrhage: population-based autopsy study
Source: Acta Neuropathol. 2026 Jan 24;151(1):8. doi: 10.1007/s00401-026-02980-0 (PMC12831695; doi:10.1007/s00401-026-02980-0)
Supplement: Supplementary file 2 — Supplementary file2 (DOCX 2949 KB) [file 401_2026_2980_MOESM2_ESM.docx]

**Supplementary Material 2**

**Pathological insights into cerebral amyloid angiopathy underlying intracerebral haemorrhage: population-based autopsy study**

**Contents**

[**Supplementary Method 1: Neuropathological assessment** 3](#_Toc217681767)

[**Supplementary Method 2: Definitions of regional and global CAA severity** 7](#_Toc217681768)

[**Supplementary Fig. 1. Study flow chart** 8](#_Toc217681769)

[**Supplementary Fig. 2. Bubble plots of CAA severity in ICH-affected hemisphere versus ICH-unaffected hemisphere, and in ICH-affected lobe versus contralateral homologous ICH-unaffected lobe** 9](#_Toc217681770)

[**Supplementary Fig. 3. The regional distribution of CAA presence and its severity in first-ever lobar ICH, stratified by age tertiles** 10](#_Toc217681771)

[**Supplementary Fig. 4. The regional distribution of CAA presence and its severity in first-ever lobar ICH, stratified by *APOE* ε2 or ε4 allele possession** 11](#_Toc217681772)

[**Supplementary Fig. 5. The regional distribution of CAA presence and its severity in first-ever lobar ICH, stratified by Thal phases and Braak stages** 12](#_Toc217681773)

[**Supplementary Table 1. Comparisons of clinical and imaging features in LINCHPIN participants who underwent autopsy versus those who did not** 13](#_Toc217681774)

[**Supplementary Table 2. Distribution of CAA pathology in first-ever lobar ICH** 14](#_Toc217681775)

[**Supplementary Table 3. Pairwise comparisons of CAA and vasculopathy scores across five brain regions** 15](#_Toc217681776)

[**Supplementary Table 4. Cross-tabulations of the Vonsattel grade of CAA in the lobe containing the ICH epicentre against the global cerebral parenchymal CAA severity in lobar ICH, stratified by age tertiles at index ICH** 16](#_Toc217681777)

[**Supplementary Table 5. Cross-tabulations of the Vonsattel grade of CAA in the lobe containing the ICH epicentre against definite CAA in lobar ICH, stratified by age tertiles at index ICH** 17](#_Toc217681778)

[**Supplementary Table 6. Diagnostic accuracy of the simulated cortical biopsy for identifying CAA presence in lobar ICH stratified by age tertiles** 18](#_Toc217681779)

[**Supplementary Table 7. Cross-tabulations of CAA and vasculopathy severity in the lobe containing the ICH epicentre against the global cerebral CAA and vasculopathy severity in lobar ICH** 19](#_Toc217681780)

[**Supplementary Table 8. Cross-tabulations of the Vonsattel grade of CAA in the lobe containing the ICH epicentre against the global cerebral parenchymal CAA severity and definite CAA, in lobar ICH with autopsy performed within one year after index ICH (n=67)** 20](#_Toc217681781)

[**Supplementary Table 9. Diagnostic accuracy of the simulated cortical biopsy for identifying CAA presence in lobar ICH with autopsy performed within one year after index ICH** 21](#_Toc217681782)

[**Supplementary Table 10. Cross-tabulations of CAA and vasculopathy severity in the lobe containing the ICH epicentre against the global cerebral CAA and vasculopathy severity in lobar ICH with autopsy performed within one year after index ICH (n=67)** 22](#_Toc217681783)

[**Supplementary Table 11. Diagnostic accuracy of the simulated cortical biopsy for identifying CAA and vasculopathy severity in lobar ICH with autopsy performed within one year after index ICH** 23](#_Toc217681784)

[**References** 24](#_Toc217681785)

## **Supplementary Method 1: Neuropathological assessment**

LINCHPIN (Lothian IntraCerebral Haemorrhage, Pathology, Imaging and Neurological Outcome study) research autopsies were conducted by a neuropathologist in accordance with a standard procedure.^1^ The maximum interval from death to autopsy was 5 days.

The brain was weighed, the cerebellum and brainstem were removed, and 1 cm thick coronal sections were made. The cerebellum was sliced sagittally and the brainstem axially. Tissue samples approximately 2 × 2 × 1 cm were taken from defined neuroanatomical areas: anterior frontal parasagittal cortex (BA9), Broca’s area (BA) 44/45, temporal tip (BA38), caudate nucleus, basal ganglia, hippocampus, thalamus, and frontal, temporal, parietal, and occipital white matter, as well as the cerebellum, pons, and medulla. Each sample was bisected in the same plane as it was cut; one piece was placed in a plastic cassette and ﬁxed in 10% unbuffered formalin in a plastic tube for 24 - 72 hours before histology evaluation. The complementary sample was frozen in nitrogen vapor for long-term storage at -150°C to support future research applications.

Tissue from the left cerebral hemisphere only was used for other small vessel disease (SVD) (non-cerebral amyloid angiopathy [CAA] SVD) evaluation, as SVD was usually considered symmetrical. The right hemisphere was also studied in 10% of cases, randomly chosen, to ensure this symmetry also applied to our population.

For SVD assessment, CAA was graded at autopsy using the Love scale (**Table I, Figure I**) and defined in simulated biopsy according to the Vonsattel scale (**Table II**). Non-amyloid SVD was rated on a 4-point scale (**Table III**). Neurodegenerative pathology was evaluated by Thal phase for *β*-amyloid (A*β*) plaques and Braak stage for neurofibrillary tangles (**Tables IV–V**).

**Figure I. Example images of histopathological grading for CAA using Love scale.**

**
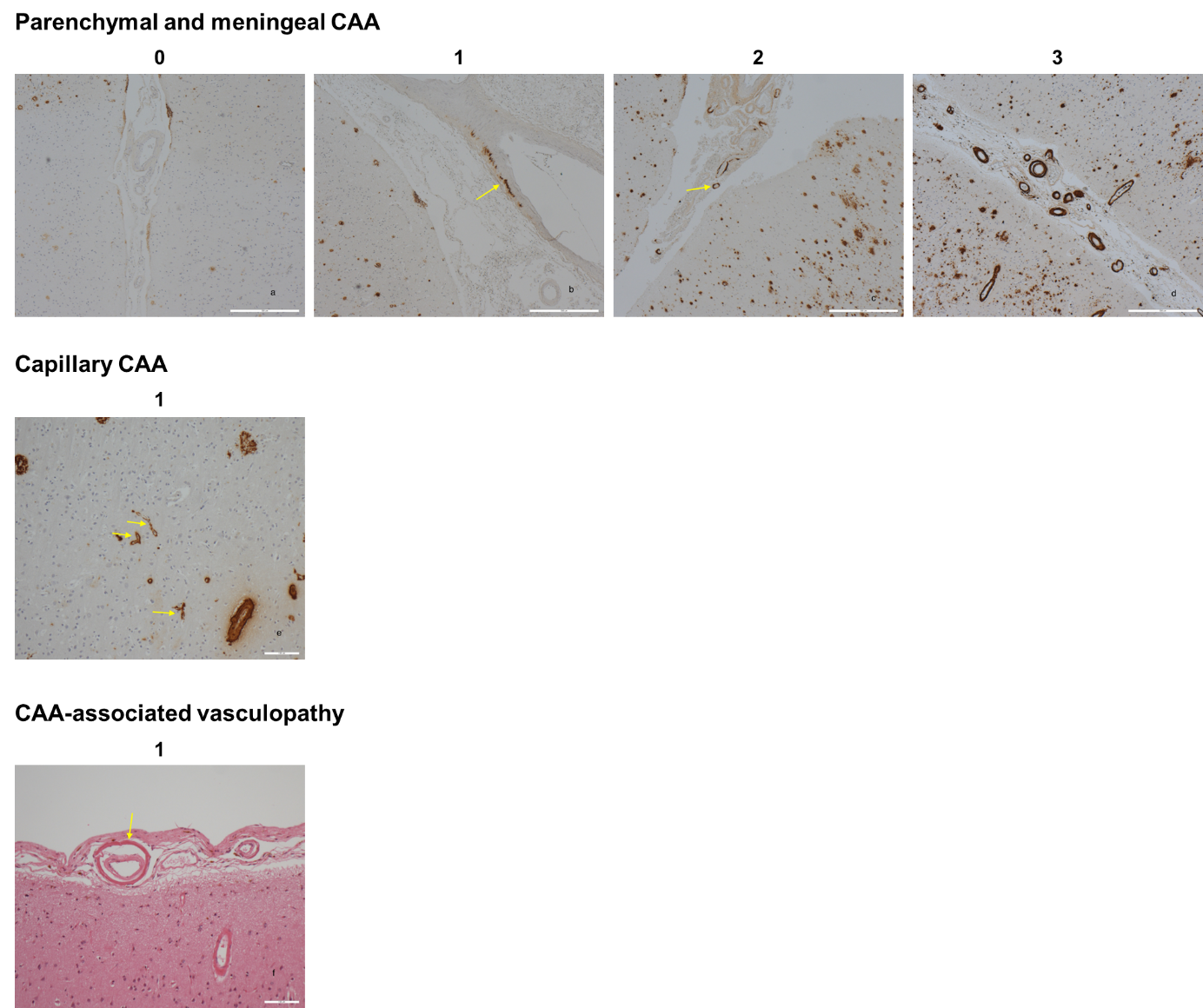
**

CAA was assessed using the criteria described by Love *et al*.^2^ a) Normal vessels within the subarachnoid space and brain parenchyma show no *β*-amyloid deposition [4G8 immunohistochemistry, x40]; b) In grade 1 most areas studied in a single section lacked any *β*-amyloid deposition, but focal vessel wall deposition can be seen (arrow) [4G8 immunohistochemistry, x40]. c) In grade 2 several vessels show *β*-amyloid deposition, and in one vessel (arrow) this is circumferential [4G8 immunohistochemistry, x40]. d) In grade 3 many parenchymal and leptomeningeal vessels show circumferential *β*-amyloid deposition [4G8 immunohistochemistry, x40]. e) The presence of any capillary CAA (arrows) scores 1 point [4G8 immunohistochemistry, x100]. f) Vasculopathy, in this example determined by concentric splitting of the vessel wall (arrows) scores 1 point [H&E stained section, x100]. CAA = cerebral amyloid angiopathy.

**Table I. Love scale for grading CAA and CAA-associated vasculopathy in post-mortem brain tissue.^2^**

| **CAA severity** | **Parenchymal CAA** | **Meningeal CAA** | **Capillary CAA** | **Vasculopathy** |
| --- | --- | --- | --- | --- |
| 0 | Absent | Absent | Absent | Absent |
| 1 | Scant A*β* deposition | Scant A*β* deposition | Present | Occasional vessel |
| 2 | Some circumferential A*β* | Some circumferential A*β* |  | Many vessels |
| 3 | Widespread circumferential A*β* | Widespread circumferential A*β* |  |  |

This method is scored separately in each cerebral (frontal, temporal, parietal, and occipital) and cerebellar lobe.

**Table II. Vonsattel scale for grading CAA.^3, 4^**

| **CAA severity** | **Features** |
| --- | --- |
| 0 | Absence of A*β* deposition in vessels |
| 1 | Presence of patchy A*β* deposition in an otherwise normal-appearing vessel |
| 2 | Complete replacement of the media by A*β* with the thickened vessel wall |
| 3 | Complete replacement of the media by A*β* and cracking of the vessel wall (creating a vessel-within-vessel appearance) affecting at least 50% of the circumference of the vessel |
| 4 | Presence of an A*β*-laden vessel of scarring and fibrinoid necrosis |

This method scores the most advanced degree of parenchymal or meningeal CAA present within the specimen.

**Table III. Scale for grading non-amyloid SVD.^5^**

| **Other SVD severity** | **Features** |
| --- | --- |
| 0 | Very mild, occasional arteriolosclerosis without media splitting or luminal narrowing |
| 1 | Widespread mild or focal moderate arteriolosclerosis |
| 2 | Widespread moderate or focal severe arteriolosclerosis, with splitting of the media and narrowing of the lumen |
| 3 | Widespread severe arteriolosclerosis, fibrinoid necrosis, lipohyalinosis, evidence of vascular occlusion with or without recanalisation |

This method is scored in frontal, central, periventricular, and occipital white matter, basal ganglia at level of mammillary body, and thalamus at level of lateral geniculate body in the left hemisphere, as SVD is usually considered symmetrical.^6^

**Table IV. Thal phase for grading Aβ plaques.^7^**

| **Thal phase** | **Features** |
| --- | --- |
| 0 | Absence of A*β* plaques |
| 1 | A*β* deposit in the frontal, parietal, temporal, or occipital neocortex |
| 2 | Additional A*β* deposit in the allocortex |
| 3 | Additional A*β* deposit in the diencephalic nuclei and striatum |
| 4 | Additional A*β* deposit in distinct brainstem nuclei (substantia nigra, red nucleus, central gray, superior and inferior collicle, inferior olivary nucleus, intermediate reticular zone) |
| 5 | Additional A*β* deposit in the cerebellum and additional brainstem nuclei (pontine nuclei, locus coeruleus, parabrachial nuclei, reticulo-tegmental nucleus, dorsal tegmental nucleus, and oral and central raphe nuclei) |

**Table V. Braak stage for grading neurofibrillary tangles.^8^**

| **Braak stage** | **Features** |
| --- | --- |
| 0 | No neurofibrillary tangles |
| I | Modest neurofibrillary tangles confined to the transentorhinal cortex |
| II | Numerous neurofibrillary tangles confined to the transentorhinal cortex |
| III | Severe involvement of layer Pre-α in both the transentorhinal and entorhinal regions, with only a few neurofibrillary tangles in layers Pri-α and Pre-β, and modest involvement of the hippocampal formation |
| IV | Severe involvement of layer Pre-α and considerable involvement of layers Pri-α and Pre-β, plus numerous neurofibrillary tangles in the hippocampal formation |
| V | More severe involvement of layers Pri-α, Pre-β and Pre-γ. All components of the hippocampal formation are involved, and the isocortex is severely affected |
| VI | Severe involvement of the isocortex, with neurofibrillary tangles in the extrapyramidal system |

## **Supplementary Method 2: Definitions of regional and global CAA severity**

The regional CAA severity was defined in **Table VI** according to the Love CAA rating scale.^2^

However, there is no consensus approach for deriving a global cerebral CAA stage from the Love CAA rating scale.^2^ We selected these cut-offs based on the summed original region-specific scores for each lobe and on the distribution of global CAA scores in the LINCHPIN brain bank (**Figure II**). We assigned global CAA burden categories as follows. For parenchymal and meningeal CAA, scores were categorised as 0 = none, 1-8 = mild, 9-16 = moderate, and 17-24 = severe. For capillary CAA, scores were categorised as 0 = absent and 1-8 = present. For CAA-associated vasculopathy, scores were categorised as 0 = none, 1-8 = mild-to-moderate, and 9-16 = severe.

**Table VI. Definitions for regional CAA severity**

| **CAA severity** | **Parenchymal CAA** | **Meningeal CAA** | **Capillary CAA** | **Vasculopathy** |
| --- | --- | --- | --- | --- |
| 0 | None | None | Absent | None |
| 1 | Mild | Mild | Present | Mild-to-moderate |
| 2 | Moderate | Moderate |  | Severe |
| 3 | Severe | Severe |  |  |

**Figure II. Distribution of the summed global CAA scores in first-ever ICH participants with autopsy**

**
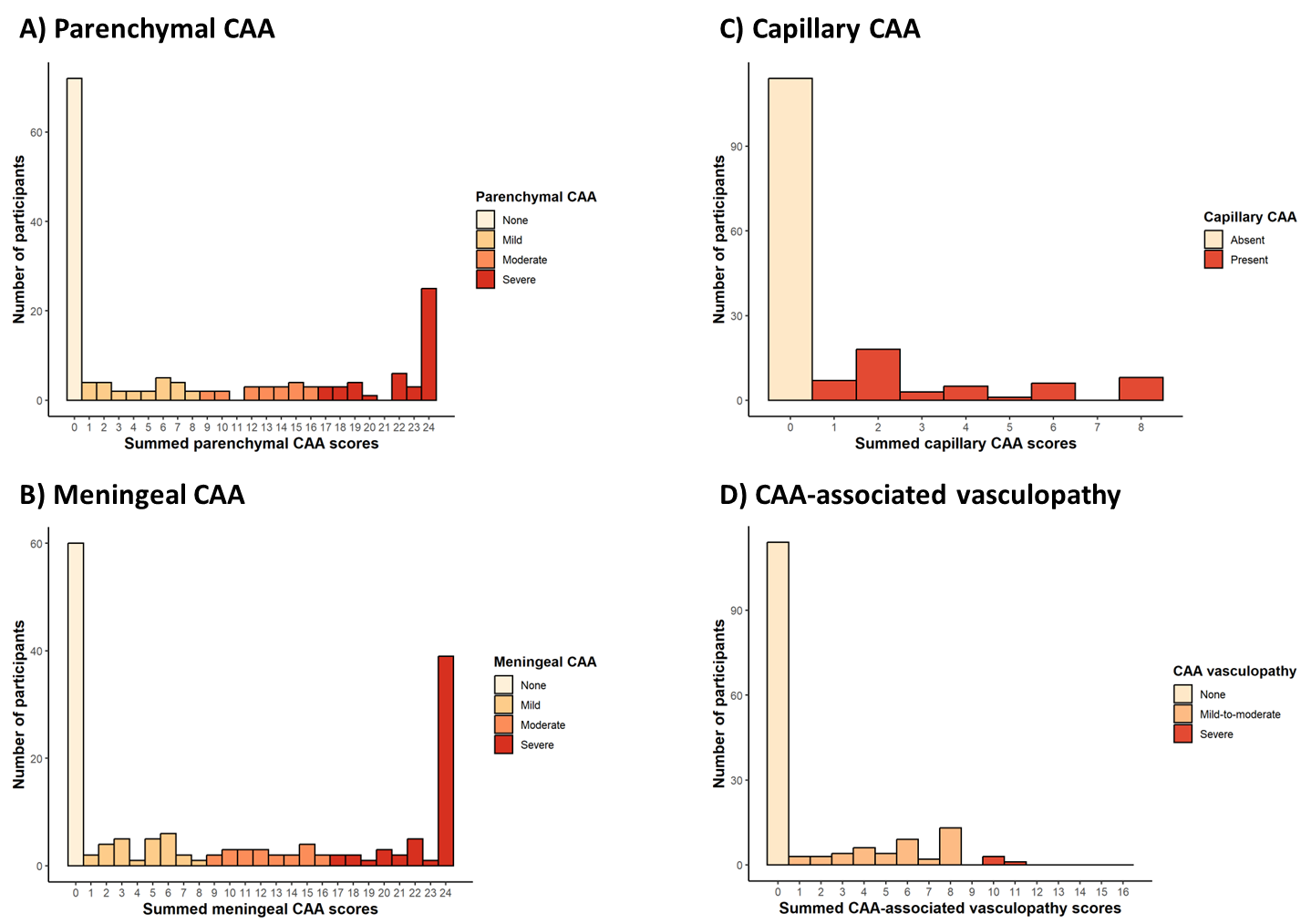
**

## **Supplementary Fig. 1. Study flow chart**

**
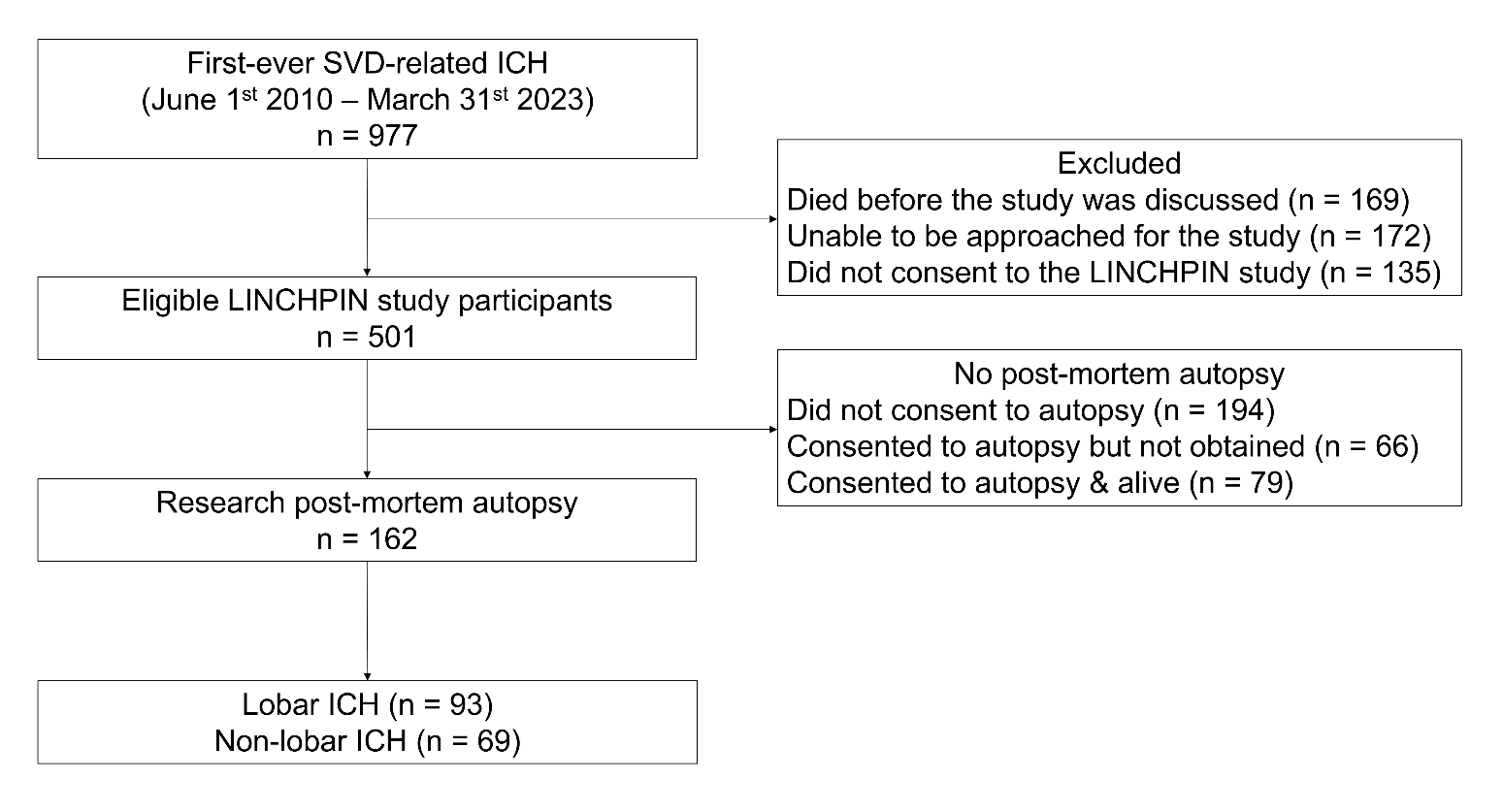
**

SVD = small vessel disease; ICH = intracerebral haemorrhage.

## **Supplementary Fig. 2. Bubble plots of CAA severity in ICH-affected hemisphere versus ICH-unaffected hemisphere, and in ICH-affected lobe versus contralateral homologous ICH-unaffected lobe**


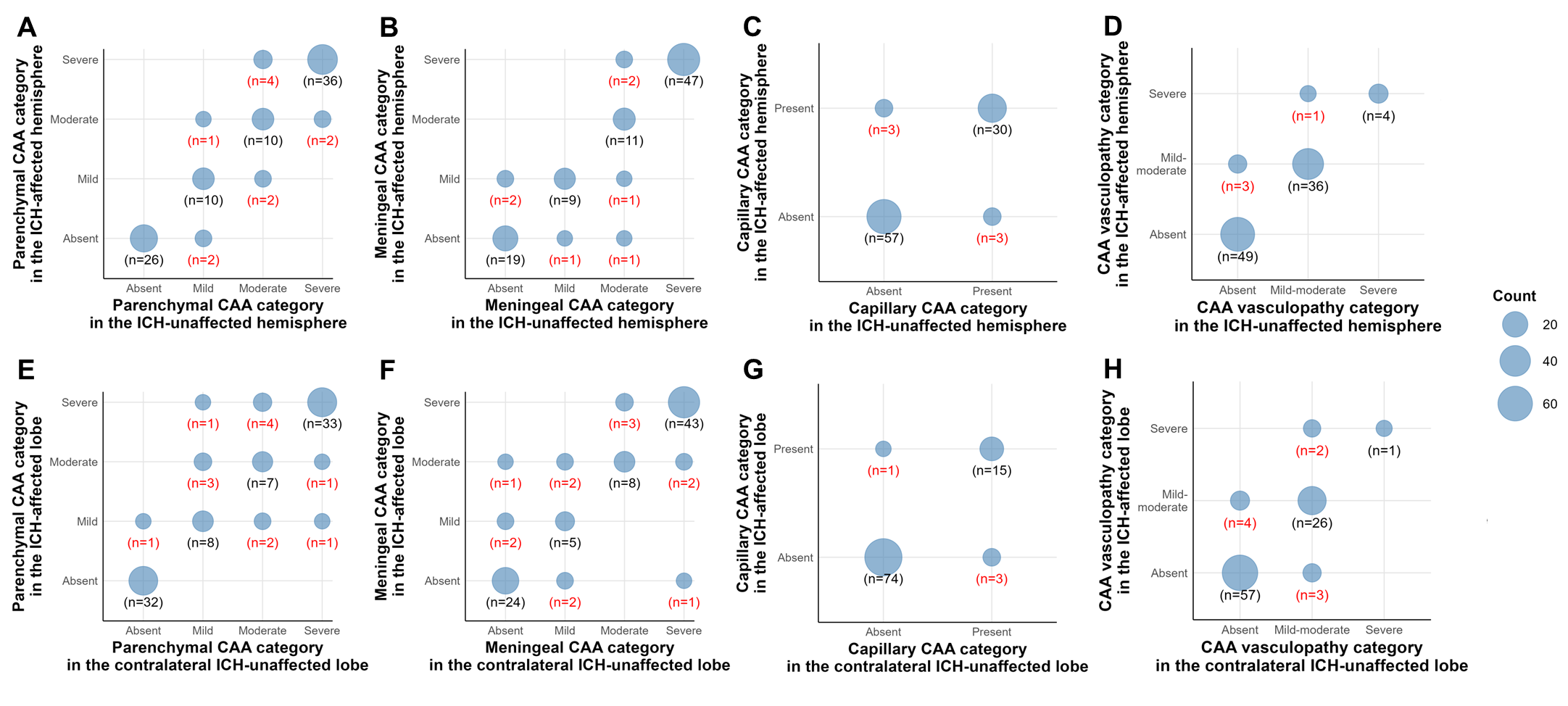


There are no significant differences of the parenchymal CAA (A, E), meningeal CAA (B, F), capillary CAA (C, G), and CAA vasculopathy (D, H) severity in ICH-affected versus ICH-unaffected hemisphere (top panel), and in ICH-affected versus contralateral homologous ICH-unaffected lobe (bottom panel) in 93 participants with lobar ICH. CAA = cerebral amyloid angiopathy; ICH = intracerebral haemorrhage.

## **Supplementary Fig. 3. The regional distribution of CAA presence and its severity in first-ever lobar ICH, stratified by age tertiles**


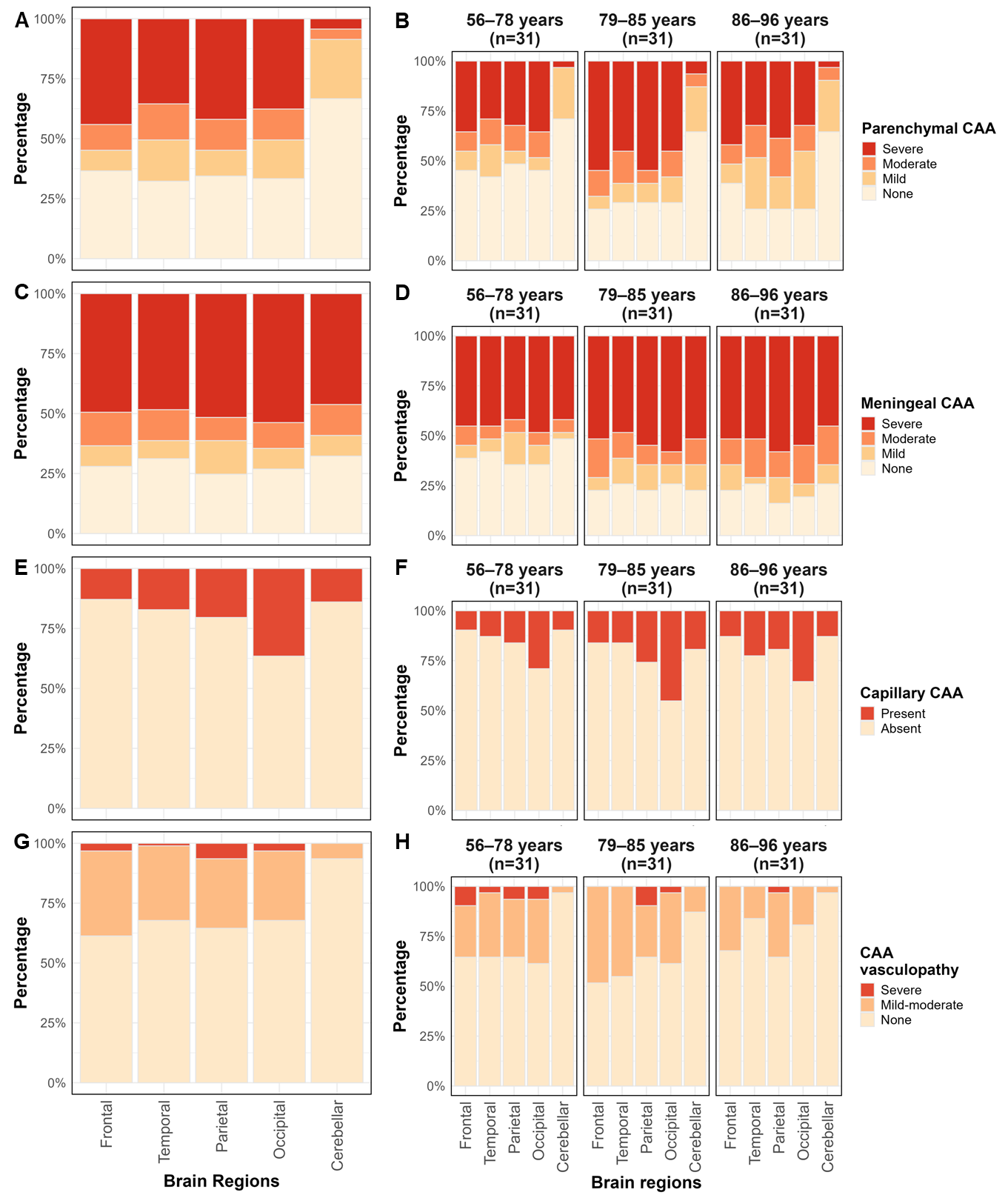


The presence and severity of parenchymal CAA (A-B), meningeal CAA (C-D), capillary CAA (E-F), and CAA-associated vasculopathy (G-H) are shown. We assess the severity of CAA and vasculopathy pathology separately in frontal, temporal, parietal, occipital, and cerebellar lobes in participants with lobar ICH (n=93, left column). There is no occipital predominance for parenchymal CAA, meningeal CAA, or CAA-associated vasculopathy, whereas capillary CAA demonstrates an occipital predominance. The severity and distribution pattern of CAA pathology do not differ across subgroups stratified by age tertiles at the time of the index ICH (right column). CAA = cerebral amyloid angiopathy; ICH = intracerebral haemorrhage.

## **Supplementary Fig. 4. The regional distribution of CAA presence and its severity in first-ever lobar ICH, stratified by *APOE* ε2 or ε4 allele possession**


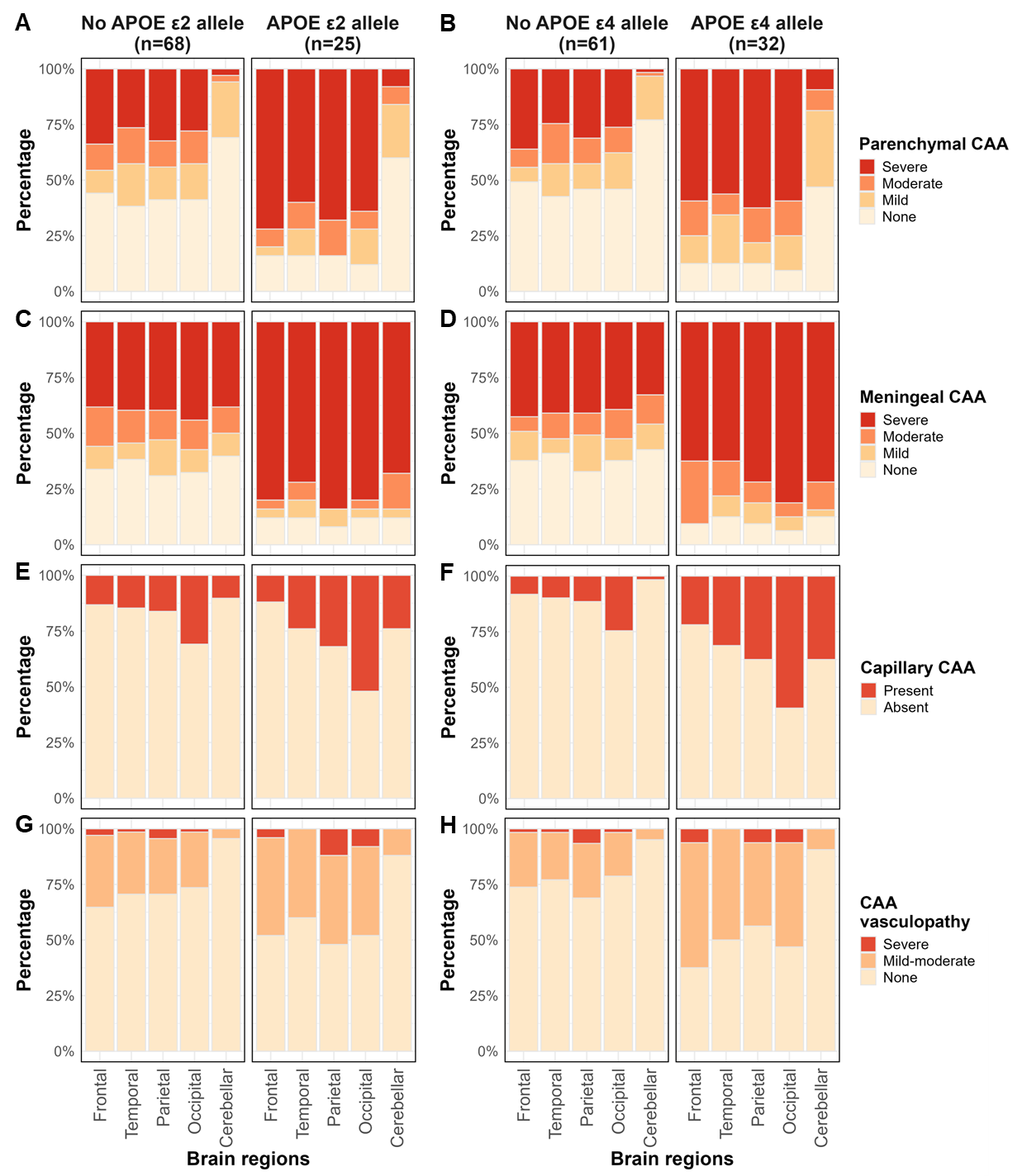


The presence and severity of parenchymal CAA (A-B), meningeal CAA (C-D), capillary CAA (E-F), and CAA-associated vasculopathy (G-H) are shown. The CAA and vasculopathy scores are higher in carriers of *APOE* ε2 (left column) or ε4 (right column) alleles compared with non-carriers. The occipital predominance restricted to capillary CAA pathology remains consistent across subgroups stratified by *APOE* ε2 or ε4 allele. CAA = cerebral amyloid angiopathy; ICH = intracerebral haemorrhage.

## **Supplementary Fig. 5. The regional distribution of CAA presence and its severity in first-ever lobar ICH, stratified by Thal phases and Braak stages**


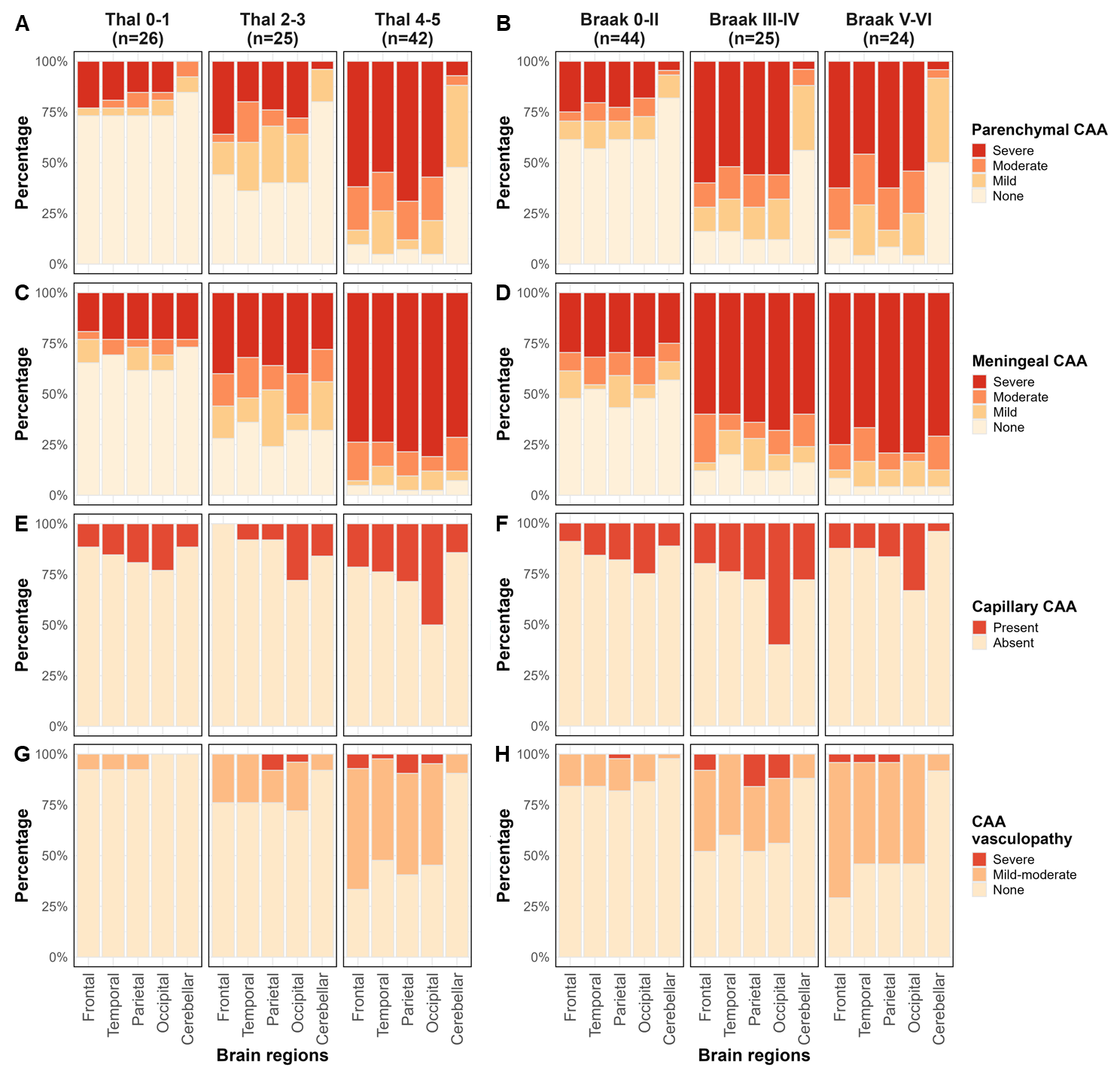


The presence and severity of parenchymal CAA (A-B), meningeal CAA (C-D), capillary CAA (E-F), and CAA-associated vasculopathy (G-H) are shown. The CAA and vasculopathy scores increase significantly with higher Thal phase for amyloid plaques (left column) and Braak stage for neurofibrillary tangles (right column), except for the severity of capillary CAA which does not differ across Braak stages. The occipital predominance restricted to capillary CAA pathology remains consistent across subgroups stratified by Thal phase and Braak stage. CAA = cerebral amyloid angiopathy; ICH = intracerebral haemorrhage.

## **Supplementary Table 1. Comparisons of clinical and imaging features in LINCHPIN participants who underwent autopsy versus those who did not**

| **LINCHPIN cohort**  **(n = 501)** | **LINCHPIN participants who underwent autopsy**  **(n = 162)** | **LINCHPIN participants who did not undergo autopsy^#^**  **(n = 339)** | ***P* value** |
| --- | --- | --- | --- |
| Sex, male | 79 (48.8) | 181 (53.4) | 0.332 |
| Age at index ICH, years | 81 (75, 87) | 74 (62, 81) | **<0.001** |
| **Co-morbidities before ICH** |  |  |  |
| Hypertension | 110 (67.9) | 207 (61.1) | 0.137 |
| Diabetes mellitus | 24 (14.8) | 39 (11.5) | 0.296 |
| Ischaemic stroke^*^ | 28 (17.3) | 38 (11.2) | 0.062 |
| Atrial fibrillation^*^ | 44 (27.2) | 52 (15.4) | **0.002** |
| Myocardial infarction | 19 (11.7) | 17 (5.0) | **0.006** |
| Cognitive status at ICH |  |  | **<0.001** |
| Unimpaired cognition | 113 (69.8) | 304 (89.7) |  |
| Mild cognitive impairment | 23 (14.2) | 17 (5.0) |  |
| Dementia | 26 (16.0) | 18 (5.3) |  |
| **Medication at ICH** |  |  |  |
| Antiplatelet drugs | 73 (45.1) | 108 (31.9) | **0.004** |
| Anticoagulant drugs | 26 (16.0) | 48 (14.2) | 0.577 |

^*^Data missing for 1 participant.

^#^This group includes 194 participants who do not consent to autopsy, 66 who consent but with no autopsy obtained, and 79 who consent but are still alive.

Data are shown as n (%) for categorical variables or median (interquartile range) for non-normally distributed continuous variables. LINCHPIN study = Lothian IntraCerebral Haemorrhage, Pathology, Imaging and Neurological Outcome study; ICH = intracerebral Haemorrhage; SVD = small vessel disease.

## **Supplementary Table 2. Distribution of CAA pathology in first-ever lobar ICH**

|  | **Frontal lobe** | **Temporal lobe** | **Parietal lobe** | **Occipital lobe** | **Cerebellum** |
| --- | --- | --- | --- | --- | --- |
| Moderate-to-severe parenchymal CAA | 51 (54.8) | 47 (50.5) | 51 (54.8) | 47 (50.5) | 8 (8.6) |
| Moderate-to-severe meningeal CAA | 59 (63.4) | 57 (61.3) | 57 (61.3) | 60 (64.5) | 55 (59.1) |
| Capillary CAA presence | 12 (12.9) | 16 (17.2) | 19 (20.4) | 34 (36.6) | 13 (14.0) |
| CAA-associated vasculopathy presence | 36 (38.7) | 30 (32.3) | 33 (35.5) | 30 (32.3) | 6 (6.5) |

Data are shown as n (%) for categorical variables. CAA = cerebral amyloid angiopathy; ICH = intracerebral haemorrhage.

## **Supplementary Table 3. Pairwise comparisons of CAA and vasculopathy scores across five brain regions**

| **Comparisons**  **(Region A vs. region B)** | **Parenchymal CAA** | | **Meningeal CAA** | | **Capillary CAA** | | **CAA-associated vasculopathy** | |
| --- | --- | --- | --- | --- | --- | --- | --- | --- |
|  | **Odds ratio (95% CI)** | ***P* value** | **Odds ratio (95% CI)** | ***P* value** | **Odds ratio (95% CI)** | ***P* value** | **Odds ratio (95% CI)** | ***P* value** |
| Frontal vs. temporal | 1.15 (0.99-1.33) | 0.143 | 1.10 (0.91-1.33) | 0.495 | 0.71 (0.45-1.14) | 0.221 | 1.39 (1.05-1.83) | 0.041^*^ |
| Frontal vs. parietal | 1.00 (0.84-1.19) | 0.980 | 1.00 (0.84-1.18) | 0.957 | 0.58 (0.35-0.96) | 0.065 | 1.03 (0.68-1.55) | 0.904 |
| Frontal vs. occipital | 1.13 (0.93-1.37) | 0.299 | 0.92 (0.75-1.13) | 0.495 | 0.26 (0.15-0.44) | <0.001^***^ | 1.29 (0.87-1.92) | 0.259 |
| Frontal vs. cerebellar | 5.88 (3.97-8.70) | <0.001^***^ | 1.19 (0.96-1.47) | 0.270 | 0.91 (0.53-1.57) | 0.739 | 9.42 (4.27-20.80) | <0.001^***^ |
| Temporal vs. parietal | 0.88 (0.71-1.08) | 0.299 | 0.90 (0.76-1.07) | 0.443 | 0.81 (0.56-1.16) | 0.318 | 0.75 (0.49-1.15) | 0.259 |
| Temporal vs. occipital | 0.99 (0.81-1.20) | 0.980 | 0.84 (0.70-1.01) | 0.205 | 0.36 (0.22-0.59) | <0.001^***^ | 0.94 (0.62-1.41) | 0.838 |
| Temporal vs. cerebellar | 6.05 (4.09-8.94) | <0.001^***^ | 1.08 (0.88-1.32) | 0.495 | 1.28 (0.72-2.28) | 0.450 | 7.00 (3.10-15.84) | <0.001^***^ |
| Parietal vs. occipital | 1.13 (0.92-1.38) | 0.299 | 0.93 (0.78-1.11) | 0.495 | 0.45 (0.29-0.69) | 0.001^**^ | 1.26 (0.89-1.79) | 0.259 |
| Parietal vs. cerebellar | 6.15 (4.17-9.07) | <0.001^***^ | 1.21 (1.01-1.45) | 0.170 | 1.58 (0.91-2.76) | 0.178 | 8.69 (4.10-18.42) | <0.001^***^ |
| Occipital vs. cerebellar | 5.91 (4.03-8.64) | <0.001^***^ | 1.29 (1.06-1.58) | 0.109 | 3.55 (2.13-5.90) | <0.001^***^ | 7.20 (3.32-15.62) | <0.001^***^ |

Odds ratios (95% CI) show the relative likelihood of higher scores in Region A compared with Region B for each pairwise comparison using generalised estimating equations. *P* values are adjusted for multiple comparisons using false discovery rate correction. ^*^*P* < 0.05, ^**^*P* < 0.01, ^***^*P* < 0.001. CAA = cerebral amyloid angiopathy; CI = confidence interval.

## **Supplementary Table 4. Cross-tabulations of the Vonsattel grade of CAA in the lobe containing the ICH epicentre against the global cerebral parenchymal CAA severity in lobar ICH, stratified by age tertiles at index ICH**

| **All participants** | | | | **All participants** | | | |
| --- | --- | --- | --- | --- | --- | --- | --- |
| **Cerebral lobe affected by ICH (Index test)** | **Global parenchymal CAA**  **(Reference standard)** | |  | **Cerebral lobe affected by ICH (Index test)** | **Global parenchymal CAA**  **(Reference standard)** | |  |
|  | None/mild | Moderate/severe | Total |  | None/mild | Moderate/severe | Total |
| Vonsattel grade 0 | 27 | 0 | 27 | Vonsattel grade <2 | 31 | 2 | 33 |
| Vonsattel grade ≥1 | 12 | 54 | 66 | Vonsattel grade ≥2 | 8 | 52 | 60 |
| Total | 39 | 54 | 93 | Total | 39 | 54 | 93 |
| **Age at index ICH 56 - 78 years** | | | | **Age at index ICH 56 - 78 years** | | | |
| **Cerebral lobe affected by ICH (Index test)** | **Global parenchymal CAA**  **(Reference standard)** | |  | **Cerebral lobe affected by ICH (Index test)** | **Global parenchymal CAA**  **(Reference standard)** | |  |
|  | None/mild | Moderate/severe | Total |  | None/mild | Moderate/severe | Total |
| Vonsattel grade 0 | 12 | 0 | 12 | Vonsattel grade <2 | 14 | 1 | 15 |
| Vonsattel grade ≥1 | 4 | 15 | 19 | Vonsattel grade ≥2 | 2 | 14 | 16 |
| Total | 16 | 15 | 31 | Total | 16 | 15 | 31 |
| **Age at index ICH 79 - 85 years** | | | | **Age at index ICH 79 - 85 years** | | | |
| **Cerebral lobe affected by ICH (Index test)** | **Global parenchymal CAA**  **(Reference standard)** | |  | **Cerebral lobe affected by ICH (Index test)** | **Global parenchymal CAA**  **(Reference standard)** | |  |
|  | None/mild | Moderate/severe | Total |  | None/mild | Moderate/severe | Total |
| Vonsattel grade 0 | 8 | 0 | 8 | Vonsattel grade <2 | 8 | 1 | 9 |
| Vonsattel grade ≥1 | 1 | 22 | 23 | Vonsattel grade ≥2 | 1 | 21 | 22 |
| Total | 9 | 22 | 31 | Total | 9 | 22 | 31 |
| **Age at index ICH 86 - 96 years** | | | | **Age at index ICH 86 - 96 years** | | | |
| **Cerebral lobe affected by ICH (Index test)** | **Global parenchymal CAA**  **(Reference standard)** | |  | **Cerebral lobe affected by ICH (Index test)** | **Global parenchymal CAA**  **(Reference standard)** | |  |
|  | None/mild | Moderate/severe | Total |  | None/mild | Moderate/severe | Total |
| Vonsattel grade 0 | 7 | 0 | 7 | Vonsattel grade <2 | 9 | 0 | 9 |
| Vonsattel grade ≥1 | 7 | 17 | 24 | Vonsattel grade ≥2 | 5 | 17 | 22 |
| Total | 14 | 17 | 31 | Total | 14 | 17 | 31 |

The Vonsattel CAA score is graded on the most advanced degree of parenchymal or meningeal CAA within the lobar specimen. CAA = cerebral amyloid angiopathy; ICH = intracerebral haemorrhage.

## **Supplementary Table 5. Cross-tabulations of the Vonsattel grade of CAA in the lobe containing the ICH epicentre against definite CAA in lobar ICH, stratified by age tertiles at index ICH**

| **All participants** | | | | **All participants** | | | |
| --- | --- | --- | --- | --- | --- | --- | --- |
| **Cerebral lobe affected by ICH (Index test)** | **Definite CAA**  **(Reference standard)** | |  | **Cerebral lobe affected by ICH (Index test)** | **Definite CAA**  **(Reference standard)** | |  |
|  | Absent | Present | Total |  | Absent | Present | Total |
| Vonsattel grade 0 | 27 | 0 | 27 | Vonsattel grade <2 | 31 | 2 | 33 |
| Vonsattel grade ≥1 | 22 | 44 | 66 | Vonsattel grade ≥2 | 18 | 42 | 60 |
| Total | 49 | 44 | 93 | Total | 49 | 44 | 93 |
| **Age at index ICH 56 - 78 years** | | | | **Age at index ICH 56 - 78 years** | | | |
| **Cerebral lobe affected by ICH (Index test)** | **Definite CAA**  **(Reference standard)** | |  | **Cerebral lobe affected by ICH (Index test)** | **Definite CAA**  **(Reference standard)** | |  |
|  | Absent | Present | Total |  | Absent | Present | Total |
| Vonsattel grade 0 | 12 | 0 | 12 | Vonsattel grade <2 | 14 | 1 | 15 |
| Vonsattel grade ≥1 | 5 | 14 | 19 | Vonsattel grade ≥2 | 3 | 13 | 16 |
| Total | 17 | 14 | 31 | Total | 17 | 14 | 31 |
| **Age at index ICH 79 - 85 years** | | | | **Age at index ICH 79 - 85 years** | | | |
| **Cerebral lobe affected by ICH (Index test)** | **Definite CAA**  **(Reference standard)** | |  | **Cerebral lobe affected by ICH (Index test)** | **Definite CAA**  **(Reference standard)** | |  |
|  | Absent | Present | Total |  | Absent | Present | Total |
| Vonsattel grade 0 | 8 | 0 | 8 | Vonsattel grade <2 | 8 | 1 | 9 |
| Vonsattel grade ≥1 | 7 | 16 | 23 | Vonsattel grade ≥2 | 7 | 15 | 22 |
| Total | 15 | 16 | 31 | Total | 15 | 16 | 31 |
| **Age at index ICH 86 - 96 years** | | | | **Age at index ICH 86 - 96 years** | | | |
| **Cerebral lobe affected by ICH (Index test)** | **Definite CAA**  **(Reference standard)** | |  | **Cerebral lobe affected by ICH (Index test)** | **Definite CAA**  **(Reference standard)** | |  |
|  | Absent | Present | Total |  | Absent | Present | Total |
| Vonsattel grade 0 | 7 | 0 | 7 | Vonsattel grade <2 | 9 | 0 | 9 |
| Vonsattel grade ≥1 | 10 | 14 | 24 | Vonsattel grade ≥2 | 8 | 14 | 22 |
| Total | 17 | 14 | 31 | Total | 17 | 14 | 31 |

The Vonsattel CAA score is graded on the most advanced degree of parenchymal or meningeal CAA within the lobar specimen. Definite CAA is defined as moderate-to-severe parenchymal CAA plus the presence of CAA-associated vasculopathy. CAA = cerebral amyloid angiopathy; ICH = intracerebral haemorrhage.

## **Supplementary Table 6. Diagnostic accuracy of the simulated cortical biopsy for identifying CAA presence in lobar ICH stratified by age tertiles**

|  | **Age at index ICH**  **56 - 78 years (n=31)** | **Age at index ICH**  **79 - 85 years (n=31)** | **Age at index ICH**  **86 - 96 years (n=31)** |
| --- | --- | --- | --- |
| **Simulated biopsy vs. moderate-to-severe parenchymal CAA at autopsy** | | | |
| **Vonsattel ≥1** | | | |
| Sensitivity | 100.0 (78.2, 100.0) | 100.0 (84.6, 100.0) | 100.0 (80.5, 100.0) |
| Specificity | 75.0 (47.6, 92.7) | 88.9 (51.8, 99.7) | 50.0 (23.0, 77.0) |
| AUC | 0.88 (0.77, 0.99) | 0.94 (0.84, 1.00) | 0.75 (0.61, 0.89) |
| PPV | 78.9 (54.4, 93.9) | 95.7 (78.1, 99.9) | 70.8 (48.9, 87.4) |
| NPV | 100.0 (73.5, 100.0) | 100.0 (63.1, 100.0) | 100.0 (59.0, 100.0) |
| **Vonsattel ≥2** | | | |
| Sensitivity | 93.3 (68.1, 99.8) | 95.5 (77.2, 99.9) | 100.0 (80.5, 100.0) |
| Specificity | 87.5 (61.7, 98.4) | 88.9 (51.8, 99.7) | 64.3 (35.1, 87.2) |
| AUC | 0.90 (0.80, 1.00) | 0.92 (0.80, 1.00) | 0.82 (0.69, 0.95) |
| PPV | 87.5 (61.7, 98.4) | 95.5 (77.2, 99.9) | 77.3 (54.6, 92.2) |
| NPV | 93.3 (68.1, 99.8) | 88.9 (51.8, 99.7) | 100.0 (66.4, 100.0) |
| **Simulated biopsy vs. definite CAA at autopsy** | | | |
| **Vonsattel ≥1** | | | |
| Sensitivity | 100.0 (76.8, 100.0) | 100.0 (79.4, 100.0) | 100.0 (76.8, 100.0) |
| Specificity | 70.6 (44.0, 89.7) | 53.3 (26.6, 78.7) | 41.2 (18.4, 67.1) |
| AUC | 0.85 (0.74, 0.97) | 0.77 (0.64, 0.90) | 0.71 (0.59, 0.83) |
| PPV | 73.7 (48.8, 90.9) | 69.6 (47.1, 86.8) | 58.3 (36.6, 77.9) |
| NPV | 100.0 (73.5, 100.0) | 100.0 (63.1, 100.0) | 100.0 (59.0, 100.0) |
| **Vonsattel ≥2** | | | |
| Sensitivity | 92.9 (66.1, 99.8) | 93.8 (69.8, 99.8) | 100.0 (76.8, 100.0) |
| Specificity | 82.4 (56.6, 96.2) | 53.3 (26.6, 78.7) | 52.9 (27.8, 77.0) |
| AUC | 0.88 (0.76, 0.99) | 0.74 (0.59, 0.88) | 0.77 (0.64, 0.89) |
| PPV | 81.3 (54.4, 96.0) | 68.2 (45.1, 86.1) | 63.6 (40.7, 82.8) |
| NPV | 93.3 (68.1, 99.8) | 88.9 (51.8, 99.7) | 100.0 (66.4, 100.0) |

The Vonsattel CAA score is graded on the most advanced degree of parenchymal of meningeal CAA within the lobar specimen. Analyses are done for Vonsattel ≥1 or ≥2 on the lobe containing the ICH epicentre versus the reference standard of moderate-to-severe CAA or definite CAA (moderate-to-severe CAA plus the presence of CAA-associated vasculopathy) at autopsy. CAA = cerebral amyloid angiopathy; ICH = intracerebral haemorrhage; AUC = area under the receiver operating characteristic curve; PPV = positive predictive value; NPV = negative predictive value.

## **Supplementary Table 7. Cross-tabulations of CAA and vasculopathy severity in the lobe containing the ICH epicentre against the global cerebral CAA and vasculopathy severity in lobar ICH**

| **All participants** | | | |
| --- | --- | --- | --- |
| **Cerebral lobe affected by ICH (Index test)** | **Global parenchymal CAA**  **(Reference standard)** | |  |
|  | None/mild | Moderate/severe | Total |
| None/mild | 37 | 7 | 44 |
| Moderate/severe | 2 | 47 | 49 |
| Total | 39 | 54 | 93 |
| **Cerebral lobe affected by ICH (Index test)** | **Global meningeal CAA**  **(Reference standard)** | |  |
|  | None/mild | Moderate/severe | Total |
| None/mild | 30 | 4 | 34 |
| Moderate/severe | 2 | 57 | 59 |
| Total | 32 | 61 | 93 |
| **Cerebral lobe affected by ICH (Index test)** | **Global capillary CAA**  **(Reference standard)** | |  |
|  | Absent | Present | Total |
| Absent | 57 | 20 | 77 |
| Present | 0 | 16 | 16 |
| Total | 57 | 36 | 93 |
| **Cerebral lobe affected by ICH (Index test)** | **Global CAA-associated vasculopathy**  **(Reference standard)** | |  |
|  | Absent | Present | Total |
| Absent | 49 | 11 | 60 |
| Present | 0 | 33 | 33 |
| Total | 49 | 44 | 93 |

CAA = cerebral amyloid angiopathy; ICH = intracerebral haemorrhage.

## **Supplementary Table 8. Cross-tabulations of the Vonsattel grade of CAA in the lobe containing the ICH epicentre against the global cerebral parenchymal CAA severity and definite CAA, in lobar ICH with autopsy performed within one year after index ICH (n=67)**

| **Cerebral lobe affected by ICH (Index test)** | **Global parenchymal CAA**  **(Reference standard)** | |  | **Cerebral lobe affected by ICH (Index test)** | **Global parenchymal CAA**  **(Reference standard)** | |  |
| --- | --- | --- | --- | --- | --- | --- | --- |
|  | None/mild | Moderate/severe | Total |  | None/mild | Moderate/severe | Total |
| Vonsattel grade 0 | 21 | 0 | 21 | Vonsattel grade <2 | 24 | 2 | 26 |
| Vonsattel grade ≥1 | 9 | 37 | 46 | Vonsattel grade ≥2 | 6 | 35 | 41 |
| Total | 30 | 37 | 67 | Total | 30 | 37 | 67 |
| **Cerebral lobe affected by ICH (Index test)** | **Definite CAA**  **(Reference standard)** | |  | **Cerebral lobe affected by ICH (Index test)** | **Definite CAA**  **(Reference standard)** | |  |
|  | Absent | Present | Total |  | Absent | Present | Total |
| Vonsattel grade 0 | 21 | 0 | 21 | Vonsattel grade <2 | 24 | 2 | 26 |
| Vonsattel grade ≥1 | 18 | 28 | 46 | Vonsattel grade ≥2 | 15 | 26 | 41 |
| Total | 39 | 28 | 67 | Total | 39 | 28 | 67 |

The Vonsattel CAA score is graded on the most advanced degree of parenchymal or meningeal CAA within the lobar specimen. Definite CAA is defined as moderate-to-severe parenchymal CAA plus the presence of CAA-associated vasculopathy. CAA = cerebral amyloid angiopathy; ICH = intracerebral haemorrhage.

## **Supplementary Table 9. Diagnostic accuracy of the simulated cortical biopsy for identifying CAA presence in lobar ICH with autopsy performed within one year after index ICH**

| **Lobar ICH (n=67)** | **Moderate-to-severe CAA** | | **Definite CAA** | |
| --- | --- | --- | --- | --- |
|  | **Vonsattel ≥1** | **Vonsattel ≥2** | **Vonsattel ≥1** | **Vonsattel ≥2** |
| Sensitivity | 100.0 (90.5, 100.0) | 94.6 (81.8, 99.3) | 100.0 (87.7, 100.0) | 92.9 (76.5, 99.1) |
| Specificity | 70.0 (50.6, 85.3) | 80.0 (61.4, 92.3) | 53.8 (37.2, 69.9) | 61.5 (44.6, 76.6) |
| AUC | 0.85 (0.77, 0.93) | 0.87 (0.79, 0.96) | 0.77 (0.69, 0.85) | 0.77 (0.68, 0.86) |
| PPV | 80.4 (66.1, 90.6) | 85.4 (70.8, 94.4) | 60.9 (45.4, 74.9) | 63.4 (46.9, 77.9) |
| NPV | 100.0 (83.9, 100.0) | 92.3 (74.9, 99.1) | 100.0 (83.9, 100.0) | 92.3 (74.9, 99.1) |

The Vonsattel CAA score is graded on the most advanced degree of parenchymal or meningeal CAA within the lobar specimen. Analyses are done for Vonsattel ≥1 or ≥2 on the lobe containing the ICH epicentre versus the reference standard of moderate-to-severe CAA or definite CAA (moderate-to-severe CAA plus the presence of CAA-associated vasculopathy) at autopsy. CAA = cerebral amyloid angiopathy; ICH = intracerebral haemorrhage; AUC = area under the receiver operating characteristic curve; PPV = positive predictive value; NPV = negative predictive value.

## **Supplementary Table 10. Cross-tabulations of CAA and vasculopathy severity in the lobe containing the ICH epicentre against the global cerebral CAA and vasculopathy severity in lobar ICH with autopsy performed within one year after index ICH (n=67)**

| **Participants with autopsy performed within one year after index ICH** | | | |
| --- | --- | --- | --- |
| **Cerebral lobe affected by ICH (Index test)** | **Global parenchymal CAA**  **(Reference standard)** | |  |
|  | None/mild | Moderate/severe | Total |
| None/mild | 28 | 5 | 33 |
| Moderate/severe | 2 | 32 | 34 |
| Total | 30 | 37 | 67 |
| **Cerebral lobe affected by ICH (Index test)** | **Global meningeal CAA**  **(Reference standard)** | |  |
|  | None/mild | Moderate/severe | Total |
| None/mild | 23 | 4 | 27 |
| Moderate/severe | 1 | 39 | 40 |
| Total | 24 | 43 | 67 |
| **Cerebral lobe affected by ICH (Index test)** | **Global capillary CAA**  **(Reference standard)** | |  |
|  | Absent | Present | Total |
| Absent | 45 | 11 | 56 |
| Present | 0 | 11 | 11 |
| Total | 45 | 22 | 67 |
| **Cerebral lobe affected by ICH (Index test)** | **Global CAA-associated vasculopathy**  **(Reference standard)** | |  |
|  | Absent | Present | Total |
| Absent | 39 | 6 | 45 |
| Present | 0 | 22 | 22 |
| Total | 39 | 28 | 67 |

CAA = cerebral amyloid angiopathy; ICH = intracerebral haemorrhage.

## **Supplementary Table 11. Diagnostic accuracy of the simulated cortical biopsy for identifying CAA and vasculopathy severity in lobar ICH with autopsy performed within one year after index ICH**

| **Simulated biopsy vs. reference standard** | **Lobar ICH**  **(n=67)** |
| --- | --- |
| **Parenchymal CAA (moderate-to-severe vs. none-to-mild)** | |
| Sensitivity | 86.5 (71.2, 95.5) |
| Specificity | 93.3 (77.9, 99.2) |
| AUC | 0.90 (0.83, 0.97) |
| PPV | 94.1 (80.3, 99.3) |
| NPV | 84.8 (68.1, 94.9) |
| **Meningeal CAA (moderate-to-severe vs. none-to-mild)** | |
| Sensitivity | 90.7 (77.9, 97.4) |
| Specificity | 95.8 (78.9, 99.9) |
| AUC | 0.93 (0.87, 0.99) |
| PPV | 97.5 (86.8, 99.9) |
| NPV | 85.2 (66.3, 95.8) |
| **Capillary CAA (present vs. absent)** | |
| Sensitivity | 50.0 (28.2, 71.8) |
| Specificity | 100.0 (92.1, 100.0) |
| AUC | 0.75 (0.64, 0.86) |
| PPV | 100.0 (71.5, 100.0) |
| NPV | 80.4 (67.6, 89.8) |
| **CAA vasculopathy (present vs. absent)** | |
| Sensitivity | 78.6 (59.0, 91.7) |
| Specificity | 100.0 (91.0, 100.0) |
| AUC | 0.89 (0.82, 0.97) |
| PPV | 100.0 (84.6, 100.0) |
| NPV | 86.7 (73.2, 94.9) |

CAA = cerebral amyloid angiopathy; ICH = intracerebral haemorrhage; AUC = area under the receiver operating characteristic curve; PPV = positive predictive value; NPV = negative predictive value.

## **References**

1. Humphreys CA, Jansen MA, Munoz Maniega S, Gonzalez-Castro V, Pernet C, Deary IJ, et al. A protocol for precise comparisons of small vessel disease lesions between ex vivo magnetic resonance imaging and histopathology. *Int J Stroke*. 2019;14(3):310-320. doi:10.1177/1747493018799962

2. Love S, Chalmers K, Ince P, Esiri M, Attems J, Jellinger K, et al. Development, appraisal, validation and implementation of a consensus protocol for the assessment of cerebral amyloid angiopathy in post-mortem brain tissue. *Am J Neurodegener Dis*. 2014;3(1):19-32.

3. Greenberg SM, Vonsattel JP. Diagnosis of cerebral amyloid angiopathy. Sensitivity and specificity of cortical biopsy. *Stroke*. 1997;28(7):1418-1422. doi:10.1161/01.str.28.7.1418

4. Vonsattel JP, Myers RH, Hedley-Whyte ET, Ropper AH, Bird ED, Richardson EP, Jr. Cerebral amyloid angiopathy without and with cerebral hemorrhages: A comparative histological study. *Ann Neurol*. 1991;30(5):637-649. doi:10.1002/ana.410300503

5. Deramecourt V, Slade JY, Oakley AE, Perry RH, Ince PG, Maurage CA, et al. Staging and natural history of cerebrovascular pathology in dementia. *Neurology*. 2012;78(14):1043-1050. doi:10.1212/WNL.0b013e31824e8e7f

6. Wardlaw JM, Smith EE, Biessels GJ, Cordonnier C, Fazekas F, Frayne R, et al. Neuroimaging standards for research into small vessel disease and its contribution to ageing and neurodegeneration. *Lancet Neurol*. 2013;12(8):822-838. doi:10.1016/S1474-4422(13)70124-8

7. Thal DR, Rub U, Orantes M, Braak H. Phases of a beta-deposition in the human brain and its relevance for the development of ad. *Neurology*. 2002;58(12):1791-1800. doi:10.1212/wnl.58.12.1791

8. Braak H, Braak E. Neuropathological stageing of alzheimer-related changes. *Acta Neuropathol*. 1991;82(4):239-259. doi:10.1007/BF00308809
